# Enhancing engineering optimization using hybrid sine cosine algorithm with Roulette wheel selection and opposition-based learning

**DOI:** 10.1038/s41598-024-51343-w

**Published:** 2024-01-06

**Authors:** Vu Hong Son Pham, Nghiep Trinh Nguyen Dang, Van Nam Nguyen

**Affiliations:** Faculty of Civil Engineering, Ho Chi Minh City University of Technology (HCMUT), Vietnam National University (VNU-HCM), Ho Chi Minh City, Vietnam

**Keywords:** Computer science, Mechanical engineering, Civil engineering

## Abstract

Meta-heuristic algorithms distinguish themselves from conventional optimization methods owing to their intrinsic adaptability and straightforward implementation. Among them, the sine cosine algorithm (SCA) is lauded for its ability to transition seamlessly between exploration and exploitation phases throughout the optimization process. However, there exists potential for enhancing the balance that SCA maintains between exploration and exploitation. To augment the proficiency in global optimization of SCA, an innovative strategy—nSCA—that integrates the roulette wheel selection (RWS) with opposition-based learning was formulated. The robustness of nSCA was rigorously evaluated against leading-edge methods such as the genetic algorithm (GA), particle swarm optimization, moth-flame optimization, ant lion optimization, and multi-verse optimizer, as well as the foundational SCA. This evaluation included benchmarks set by both CEC 2019 and CEC 2021 test functions. Additionally, the performance of nSCA was confirmed through numerous practical optimization problems, emphasizing its effectiveness in applied settings. In all evaluations, nSCA consistently showcased superior performance compared to its evolutionary algorithm counterparts, delivering top-tier solutions for both benchmark functions and real-world optimization challenges. Given this compelling evidence, one can posit that nSCA serves as a strong candidate for addressing intricate optimization challenges found in real-world contexts, regardless of whether they are of a discrete or continuous nature.

## Introduction

### Evolutionary algorithm

In recent years, the scholarly community has increasingly turned its attention to nature-inspired optimization algorithms, recognizing their efficacy in addressing complex optimization challenges. Holland^1^ was among the pioneers, utilizing genetic algorithms (GA) to explore complex adaptive systems. He drew a compelling parallel between biological evolution and computational problem-solving. Kennedy and Eberhart^2^ put forth the particle swarm optimization (PSO), a method tailored for nonlinear function optimization. They detailed its evolution, tested it against benchmarks, applied it in neural network training, and explored its intersections with artificial life and GA. Rezaei et al.^3^ introduced the geometric mean optimizer (GMO). This new meta-heuristic technique leverages the capabilities of the geometric mean operator. When compared to other contemporary algorithms, GMO consistently exhibited superior performance in various optimization challenges. Mirjalili^4^ introduced the sine cosine algorithm (SCA), a unique population-based optimization approach. This technique, which utilizes sine and cosine functions to guide solution candidates, demonstrated its versatility in multiple tests, notably in the optimization of an aircraft wing’s cross-section. Such applications underscore its capacity to navigate challenges with constrained and unfamiliar search domains. Mirjalili et al.^5^ presented the multi-verse optimizer (MVO). Drawing inspiration from cosmological phenomena, the MVO was proficient in outperforming other well-regarded optimization methods across diverse benchmark tasks and real-world challenges. Gandomi^6^ put forth the interior search algorithm (ISA), a novel technique inspired by principles of interior design. With its efficacy pitted against other popular algorithms, ISA yielded promising results and featured a straightforward single-parameter tuning approach. Mirjalili^7^ rolled out the moth-flame optimization (MFO) algorithm. Inspired by the navigation techniques of moths, the MFO asserted its dominance across a gamut of benchmark functions and tangible engineering quandaries, such as marine propeller optimization. Mirjalili^8^ proffered the ant lion optimizer (ALO). Rooted in the predatory dynamics of antlions, this method underscored its pre-eminence across diverse test environments, spanning mathematical functions to intricate engineering challenges, such as ship propeller formulation, further solidifying its stature vis-à-vis other established algorithms.

In population-based evolutionary algorithms, the optimization technique is commonly divided into exploration and exploitation stages, irrespective of the algorithm’s specific characteristics^9,10^. The exploration phase focuses on investigating promising regions within the search area, where significant changes in directions can have a substantial impact. Conversely, the exploitation stage enables gradual adjustments in options and demonstrates the algorithm’s convergence by utilizing the solutions obtained during exploration. Finding the optimal trade-off between the exploration and exploitation phase is crucial to ensure the effectiveness of the algorithm in achieving global optimization.

Optimization techniques have wide applications across various fields. Xi et al.^11^ utilized advanced machine learning algorithms combined with novel optimization techniques to forecast the compressive strength of recycled aggregate concrete (RAC). Their findings highlighted the superior performance of the LGBM-based hybrid model and underlined the crucial role of factor interactions in shaping the mechanical properties of RAC. In another study, Zhou et al.^12^ applied three optimization algorithms to fine-tune the hyper-parameters of the support vector machine. Their aim was to predict the progress rate of tunnel-boring machines in hard rock conditions. Data from a water transfer tunnel project in Malaysia revealed that the MFO hybrid model surpassed other models in accuracy. Li et al.^13^ used support vector regression along with five optimization algorithms to estimate the mean fragment size (MFS) during blasting operations. Their analyses identified the grey wolf optimization (GWO) variant as the top performer. Additionally, their research found that the uniaxial compressive strength was the most significant factor influencing the prediction of blasting MFS. Son and Nguyen Dang^14^ introduced the MVO as a potent tool designed for time–cost optimization challenges in construction project management. Evaluations, especially on smaller benchmarks, reinforced the effectiveness of MVO over other stochastic optimization methods. In another insightful study, Son and Hieu^15^ developed a detailed model for logistics costs associated with precast concrete structures. This model, based on the activity-based costing method, also incorporated an enhanced ALO algorithm that combined OBL, mutation, and crossover strategies for optimal cost solutions. When compared to earlier models, this new approach demonstrated better performance in terms of convergence speed, accuracy, and overall cost reduction.

The continuous evolution and improvement of algorithms have piqued the interest of numerous researchers^16^. This interest arises from the acknowledgment that there is no universally applicable algorithm competent in addressing diverse optimization problems. This understanding compels researchers either to enhance the current algorithms to cater to novel challenges or to devise new ones that can competently rival their predecessors. Son and Nguyen Dang^17^ introduced the hybrid multi-verse optimizer model (hDMVO), a synthesis of the MVO and the SCA. This model is explicitly crafted to navigate discrete time–cost trade-off dilemmas encountered in construction project management. Its efficacy is particularly pronounced in large-scale projects, where it outstrips many established algorithms. Zhen et al.^18^ proposed a novel WPA-PSO hybrid algorithm. By harnessing the collective strengths of both methodologies, this amalgamated solution boasts enhanced prediction accuracy and stability, particularly when operating with sparse data, as opposed to its individual counterparts. Pham et al.^19^ ventured into logistics with an innovative hybrid swarm intelligence algorithm. The chief aim of this model is to refine dispatch schedules for ready-mix concrete trucks, fostering improved coordination between batching plants and construction locales. Teng et al.^20^ launched the grey wolf grasshopper hybrid algorithm (GWGHA). This algorithm targets the optimization of traffic light cycles, with a vision to curtail vehicle waiting durations and bolster on-time arrivals. The efficacy of this model is underpinned by the simulation of urban mobility (SUMO), which employed data from an assortment of global cities. Qiao et al.^21^ introduced a groundbreaking hybrid algorithm, fusing the lion swarm optimizer with the GA. Tasked with amplifying the stability and accuracy of carbon dioxide emission predictions, this model was rigorously tested using data spanning 1965 to 2017. Its performance in terms of optimization, convergence speed, and forecasting precision outshone other prevalent models. Long et al.^22^ brought forth the GWOCS, a hybrid algorithm blending the GWO with the cuckoo search (CS). Augmented with an OBL strategy, this algorithm adeptly extracts parameters from various solar PV models, utilizing experimental data across heterogeneous conditions. It achieves a harmonious interplay between exploration and exploitation, as evinced by its superior benchmark test outcomes. Dhiman and Kaur^23^ championed the hybrid particle swarm and spotted hyena optimizer (HPSSHO). This avant-garde optimization technique marries the PSO with the spotted hyena optimizer (SHO). It seeks to augment the hunting strategy of the SHO by integrating PSO dynamics. Its performance, as evidenced across thirteen benchmark functions and a nuanced 25-bar engineering design challenge, stands as a testament to its prowess over other metaheuristic approaches. Şenel et al.^24^ unveiled a hybrid algorithm that seamlessly integrates the robustness of both PSO and GWO. Notably, it shines in benchmark evaluations and real-world applications alike, consistently eclipsing other traditional and hybrid optimization techniques.

### Sine cosine algorithm

Since its introduction in 2016, the SCA has gained considerable popularity as an optimization method widely utilized in various domains to address a broad spectrum of problems. For instance, Zhao et al.^25^ developed a discrete version of SCA to overcome the challenge of community detection, while Banerjee and Nabi ^26^ proposed an SCA model to optimize the return trajectory phase of a reusable launch vehicle. Fatlawi et al.^27^ used SCA to determine camera positions for monitoring systems. Reddy et al. ^28^ presented a binary adaptation of SCA to determine the optimal commitment and dispatch of power-generating units while considering operational constraints. Tawhid and Savsani^29^ developed an enhanced SCA for the optimization of engineering design tasks with multiple objectives. Finally, Raut and Mishra^30^ proposed an advanced SCA modification that incorporates a load flow methodology leveraging data structures to optimize power distribution network reconfiguration tasks.

Given the diverse nature of optimization problems, it is widely acknowledged that there is no universally applicable optimization algorithm competent in addressing diverse optimization problems^16^. Cheng and Duan^31^ proposed a hybrid version that combines SCA and the cloud model to handle benchmark test functions with different dimensions. Bureerat and Pholdee^32^ developed a hybrid model that combines SCA and DE for detecting structural damage. Turgut^33^ proposed a model that integrates the SCA with the backtracking search algorithm to effectively address multi-objective problems in heat exchanger design. Bairathi and Gopalani^34^ improved SCA by integrating the opposition-based mechanism to instruct multi-layer neural networks. Qu et al.^35^ introduced an upgraded version of the SCA by incorporating a neighborhood search technique and a greedy Levy mutation. Finally, Pham and Nguyen^36^ proposed an integrated SCA version with tournament selection, OBL, and mutation and crossover methods to handle cement transport routing.

### The motivation of this study

The SCA, recognized for its simplicity, has carved a niche for itself as a preferred stochastic optimization technique across various scientific domains. Nonetheless, a prominent drawback associated with the SCA is its inclination to converge prematurely. This can be attributed to its undefined exploitation mechanism within the search area^37^. Such a limitation has spurred researchers to suggest a refined SCA framework, envisaged as a panacea to the intricacies intertwined with optimization issues.

In the subsequent section, the development and evolution of the nSCA are detailed. In “Analysis of performance” section, a thorough examination of the algorithm’s convergence properties is provided. Here, its behavior and efficacy are evaluated using benchmarks from CEC 2019 and CEC 2021. In “Practical application of nSCA” section, the robustness of the model is validated by subjecting the nSCA to a range of real-world optimization challenges, including the cantilever beam design, truss structure design, and the capacity vehicle routing problems. In “Conclusion” section, pivotal research insights are compiled, and potential avenues for future research are suggested. “Limitations” section presents the limitations identified in the nSCA.

## Novel version of sine cosine algorithm

### Roulette wheel selection (RWS)

The Roulette wheel selection (RWS) mechanism has been widely employed in the realm of optimization, being incorporated into numerous algorithms because of its inherent flexibility and adaptability. This mechanism is fundamentally based on the principle of selection probability, where entities are selected according to their performance metrics, most commonly their fitness values in genetic algorithms (GA). The visualization of RWS is likened to a roulette wheel, with slots assigned in proportion to an individual’s fitness. Individuals with higher fitness values are allocated larger slots, thereby augmenting their likelihood of selection for the subsequent generation. The dynamic character of RWS has prompted several refinements to its core structure. Efforts have been made to sharpen the selection criteria, while others have aimed to evade the issue of premature convergence. For example, the challenge of the well-known traveling salesman problem (TSP) was addressed by Yu et al.^38^, who instilled adaptability into RWS. A mechanism was introduced that dynamically modified the selection pressure, bolstering genetic diversity and ensuring continued exploration. Differential evolution (DE) has also been influenced by RWS. It was integrated into DE by Qian et al.^39^ for the purpose of mutation strategy selection, enhancing its convergence behaviour. In the domain of sentiment analysis, RWS was utilized by Pandey et al.^40^ to bolster the performance of the CS algorithm, leading to improved outcomes. The capabilities of RWS extend beyond traditional algorithms. A multi-dimensional strategy was advocated by Asghari et al.^41^, merging RWS with the whale-PSO algorithm. Their objective was to address intricate optimization challenges. The subject of parallelism in optimization has gained traction, and a significant contribution to this field was made by Lloyd and Amos^42^. Their research cantered on the efficacy of an autonomous RWS mechanism within parallel ant colony optimization (ACO). In the realm of power dispatching, a complex endeavour, a novel approach was proposed by Cheng et al.^43^. RWS was amalgamated with PSO to adeptly handle equality constraints.

### Opposition-based learning (OBL)

The opposition-based learning (OBL) approach has been widely recognized and utilized in diverse optimization applications, underscoring its versatility and efficacy. First introduced by Tizhoosh^44^ in 2005, OBL was presented as an innovative framework for computational intelligence, devised to generate complementary solutions for existing ones. Later, Wang et al.^45^ put forth a generalized OBL method, aiming to augment the efficiency of the PSO. In the context of construction project management, a balance in optimizing time, cost, and quality in multi-mode projects was achieved by Luong et al.^46^ through the application of opposition multiple objective DE. Further, a two-phase DE algorithm was developed by Cheng and Tran^47^ for multi-criteria decision-making, primarily targeting the equilibrium between time and cost in resource-constrained projects. The power systems sector has not been untouched by OBL’s influence. Shaw et al.^48^ formulated an algorithm that harnesses OBL principles and integrates a gravitational search strategy, aiming to optimize both economic and emission objectives simultaneously. Furthermore, the integration of OBL into various other optimization algorithms has been witnessed. This includes its incorporation into the grasshopper optimization algorithm (GOA) by Ewees et al.^49^, and into the salp swarm algorithm (SSA) by Tubishat et al.^50^. Surveying the literature reveals a clear trend: the integration of OBL can notably elevate the relative performance of numerous optimization algorithms across varied sectors. This positions OBL as a potent avenue warranting further exploration in upcoming research.

### Novel version of SCA (nSCA)

In the nSCA, each solution position is defined by a series of variables, which collectively form sets of solutions. These sets, together with their associated positions, are systematically organized into a matrix configuration, as illustrated in Eq. (1). In a similar manner, the matrix of opposite solutions, which are produced during the exploration phase, is delineated in Eq. (2). Such matrix formulations aid in proficient handling and assessment of solutions within the algorithm, thereby promoting efficient exploration and optimization of the search space.

1 $$S=\left[\begin{array}{ccc}\begin{array}{cc}{s}_{1}^{1}& {s}_{1}^{2}\end{array}& \dots & {s}_{1}^{d}\\ \begin{array}{cc}{s}_{2}^{1}& {s}_{2}^{2}\end{array}& \dots & {s}_{2}^{d}\\ \begin{array}{cc}\begin{array}{c}\dots \\ {s}_{N}^{1}\end{array}& \begin{array}{c}\dots \\ {s}_{N}^{2}\end{array}\end{array}& \begin{array}{c}\dots \\ \dots \end{array}& \begin{array}{c}\dots \\ {s}_{N}^{d}\end{array}\end{array}\right],$$

2 $${S}^{*}=\left[\begin{array}{ccc}\begin{array}{cc}{s}_{1}^{1*}& {s}_{1}^{2*}\end{array}& \dots & {s}_{1}^{d*}\\ \begin{array}{cc}{s}_{2}^{1*}& {s}_{2}^{2*}\end{array}& \dots & {s}_{2}^{d*}\\ \begin{array}{cc}\begin{array}{c}\dots \\ {s}_{N}^{1*}\end{array}& \begin{array}{c}\dots \\ {s}_{N}^{2*}\end{array}\end{array}& \begin{array}{c}\dots \\ \dots \end{array}& \begin{array}{c}\dots \\ {s}_{N}^{d*}\end{array}\end{array}\right].$$

**Figure 1:**
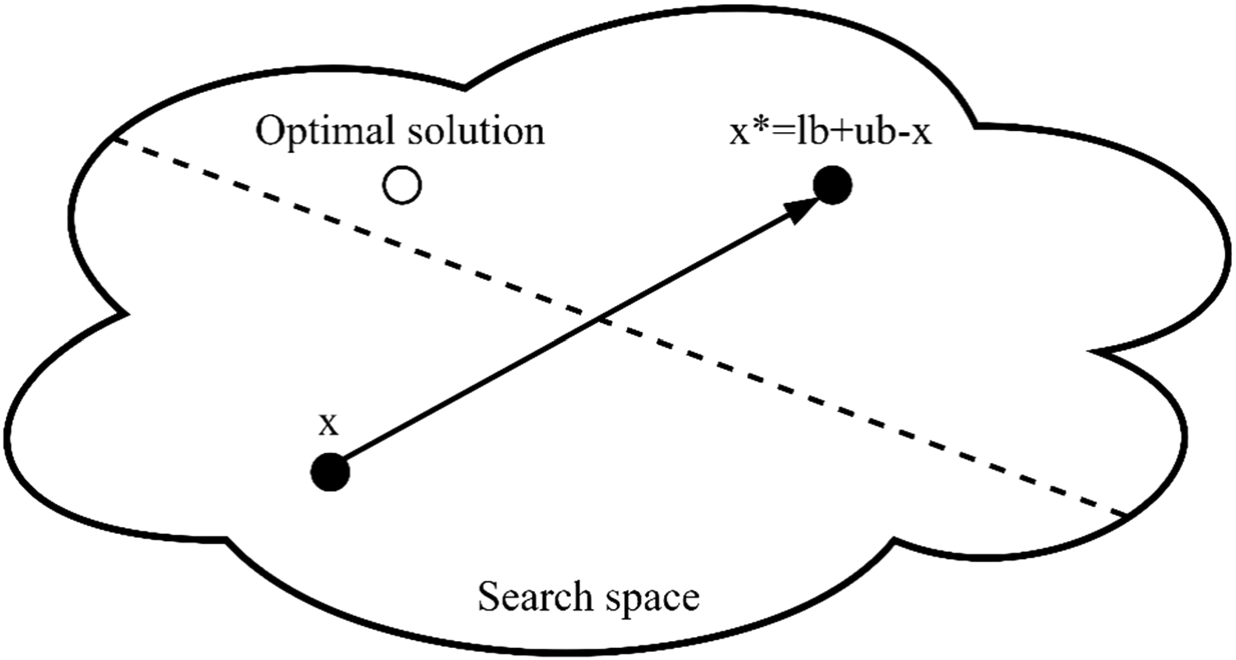
The OBL concept.

During the initial population generation phase, the OBL method, shown in Fig. 1, is employed to produce opposite solutions from those randomly generated, as illustrated in the pseudocode for nSCA in Table 1. The fitness function is subsequently applied to both the randomly generated solutions and their corresponding opposite solutions to identify the superior and inferior solutions. The superior solution is retained, while the inferior one is discarded, maintaining a consistent population size.

**Table 1:**
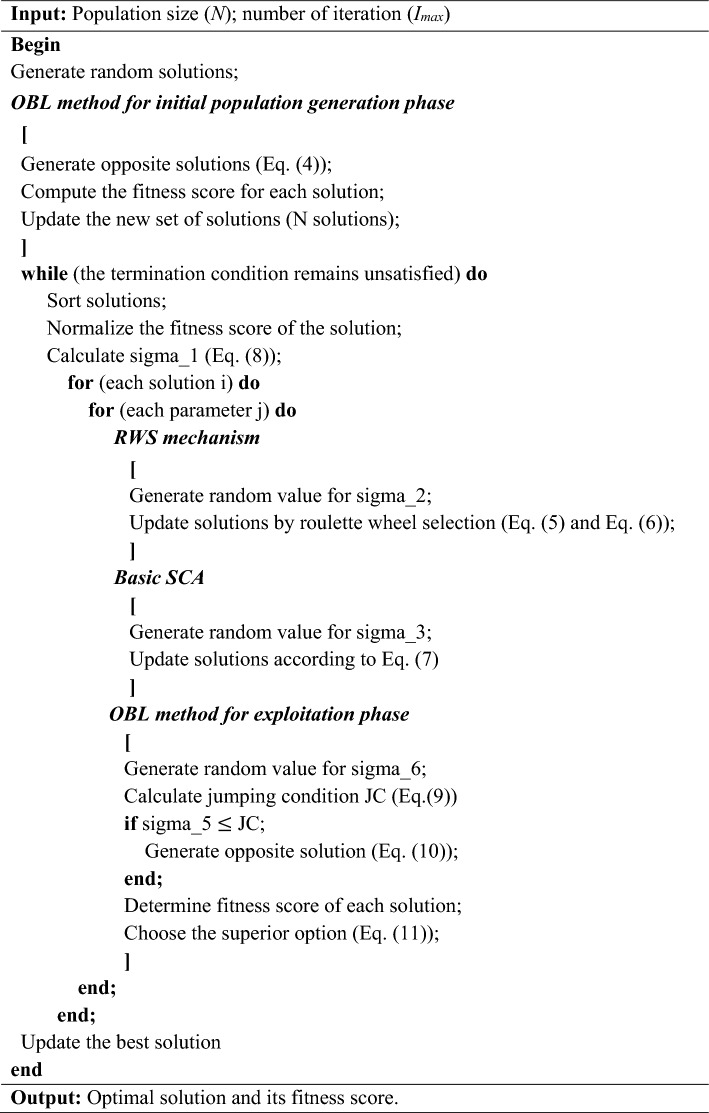
Pseudocode of the nSCA.

The opposite solution ${s}^{*}$ of the solution $s\in [{b}_{l},{b}_{u}]$ can be identified by:

3 $${s}^{*}={b}_{u}+{b}_{l}-s,$$

where *b*_*l*_ and *b*_*u*_ denote the lower and upper boundary of alternative *s*.

For a solution *S* with *d* dimensions, where each dimension is within the range of $[{b}_{l,j},{b}_{u,j}]$, an opposition solution ${S}^{*}=({s}_{1}^{*},{s}_{2}^{*},{s}_{3}^{*},\dots ,{s}_{d}^{*})$ can be characterized by:

4 $${s}_{j}^{*}={b}_{u,j}+{b}_{l,j}-{s}_{j},$$

where *b*_*l,j*_ and *b*_*u,j*_ show the minimum and maximum limits of the *j*th dimension, respectively.

**Figure 2:**
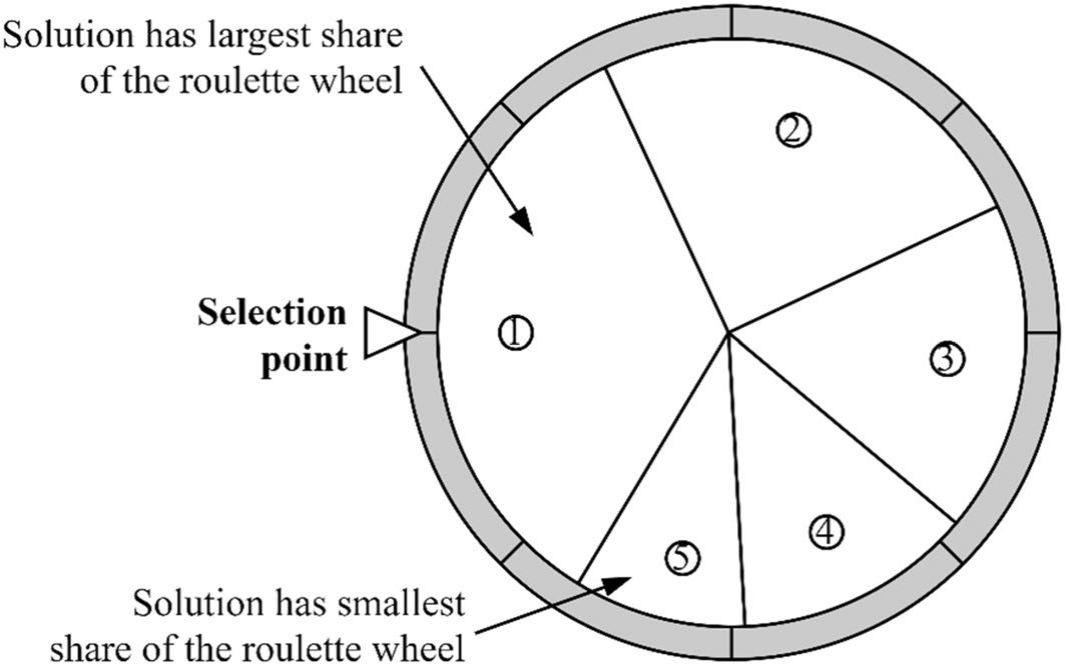
The RWS concept.

Following the update of the new solution set during the initial population generation phase, the solutions are sorted, and the current optimal solution is identified. Subsequently, the normalized fitness score for each solution is determined, playing a crucial role in the RWS mechanism, as depicted in Fig. 2. The normalized fitness score is derived using Eq. (5), and the RWS mechanism is mathematically expressed in Eq. (6). These computations and mechanisms play pivotal roles in selecting and advancing the exploration of solutions within the algorithm.

5 $$NF\left({S}_{i}\right)=\frac{F\left({S}_{i}\right)}{\sqrt[2]{\sum_{1}^{N}{F\left({S}_{i}\right)}^{2}}},$$

6 $${s}_{i}^{j}=\left\{\begin{array}{c}{s}_{1}^{j} {\sigma }_{2}<NF\left({S}_{i}\right)\\ {s}_{i}^{j} {\sigma }_{2}\ge NF\left({S}_{i}\right)\end{array}\right..$$

The terms *NF(S*_*i*_*)* and *F(S*_*i*_*)* represent the normalized fitness score and fitness score of solution *S*_*i*_, respectively. The notation ${s}_{i}^{j}$ indicates the *j*th parameter of the *i*th solution, and ${s}_{1}^{j}$ represents the *j*th parameter of the best solution achieved so far. The variable *σ*_*2*_ corresponds to a stochastic number ranging between 0 and 1. Distinctive categorization of the optimization process into exploration and exploitation stages has emerged as a focal point in earlier studies. Such a division is characteristic of many population-based stochastic methods^9^. Within the exploration phase, the optimization method employs a heightened degree of randomness, facilitating the amalgamation of solutions and quickening the identification of promising areas within the search domain. On the other hand, the exploitation phase witnesses subtle adjustments in the stochastic solutions, characterized by notably reduced stochastic fluctuations compared to the exploration phase. With regard to the SCA, mathematical formulas, encapsulated by Eq. (7), are outlined to update positions during both the exploration and exploitation phases. The value of these formulas stems from their role in directing the search trajectory of the SCA, ensuring an effective survey and utilization of the solution domain.

7 $${s}_{j}^{t+1}=\left\{\begin{array}{c}{s}_{j}^{t}+{\sigma }_{1}\times {\text{sin}}\left({\sigma }_{4}\right)\times \left|{\sigma }_{5}{P}_{j}^{t}-{s}_{j}^{t}\right| {\sigma }_{3}<0.5\\ {s}_{j}^{t}+{\sigma }_{1}\times {\text{cos}}\left({\sigma }_{4}\right)\times \left|{\sigma }_{5}{P}_{j}^{t}-{s}_{j}^{t}\right| {\sigma }_{3}\ge 0.5\end{array}\right..$$

In Eq. (7), the location of current solution in the *i*th dimension at the *t*th iteration is represented by ${s}_{j}^{t}$. The movement direction is determined by σ_1_, while *σ*_3_ is a uniformly distributed random variable between 0 and 1. Additionally, *σ*_4_ is a stochastic variable that governs the magnitude of displacement towards or away from the destination, and *σ*_5_ is a random number used as the weight for the destination. The location of the target solution in the *i*th dimension is denoted by ${D}_{j}^{t}$, and the absolute value is indicated by ||.

**Figure 3:**
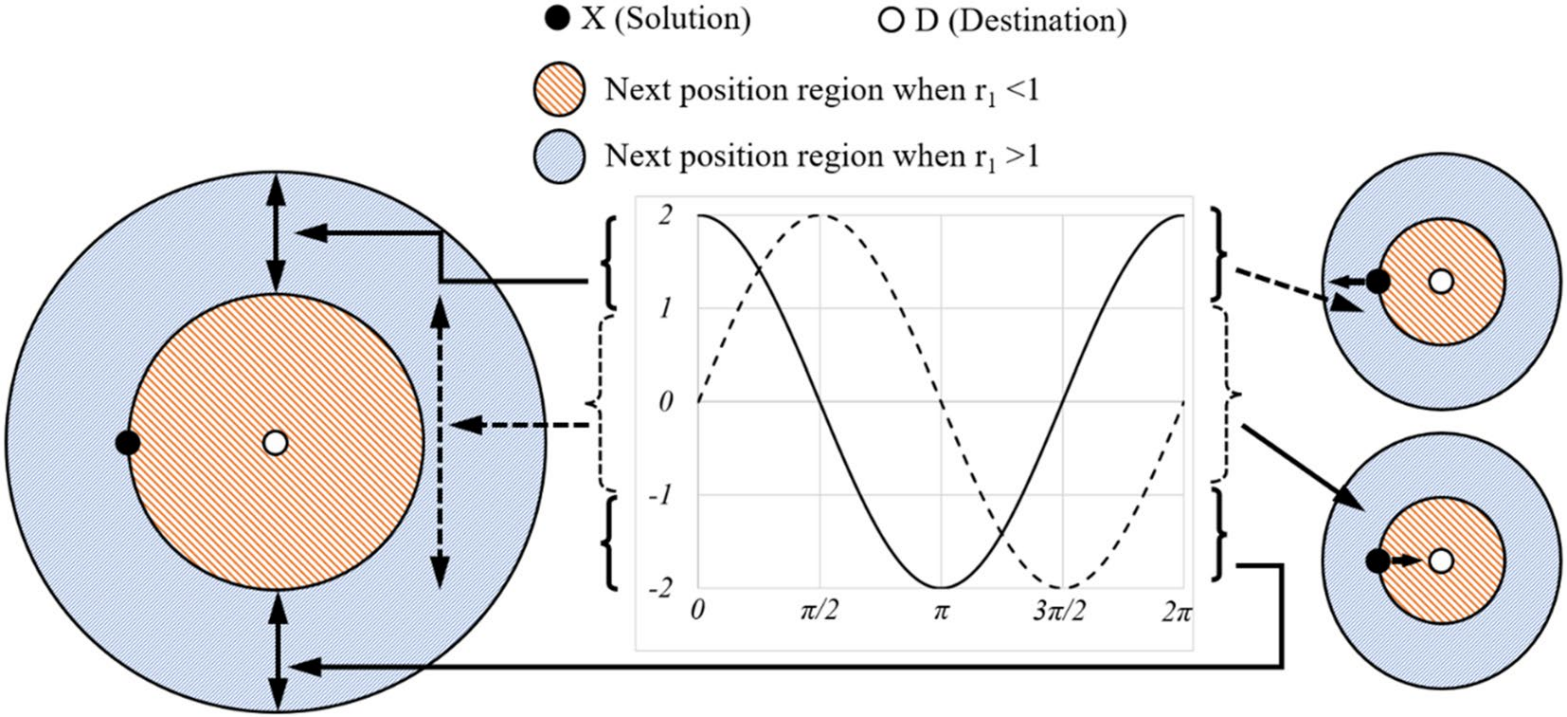
The exploration and exploitation mechanisms of the SCA.

Figure 3 presents a comprehensive model that illustrates the effectiveness of sine and cosine functions within the range of [− 2, 2]. These functions facilitate an alternative to navigate within the area bounded by them or extend beyond it, facilitating flexible movement toward the desired objective. The figure highlights the dynamic nature of the sine and cosine ranges, which are instrumental in updating the solution positions. Moreover, the inclusion of a stochastic variable, *σ*_4_, in the range of 0 to 2π, as defined in Eq. (7), introduces a stochastic element into the process. This mechanism enhances exploration within the search space, enabling a more extensive exploration of potential solutions.

**Figure 4:**
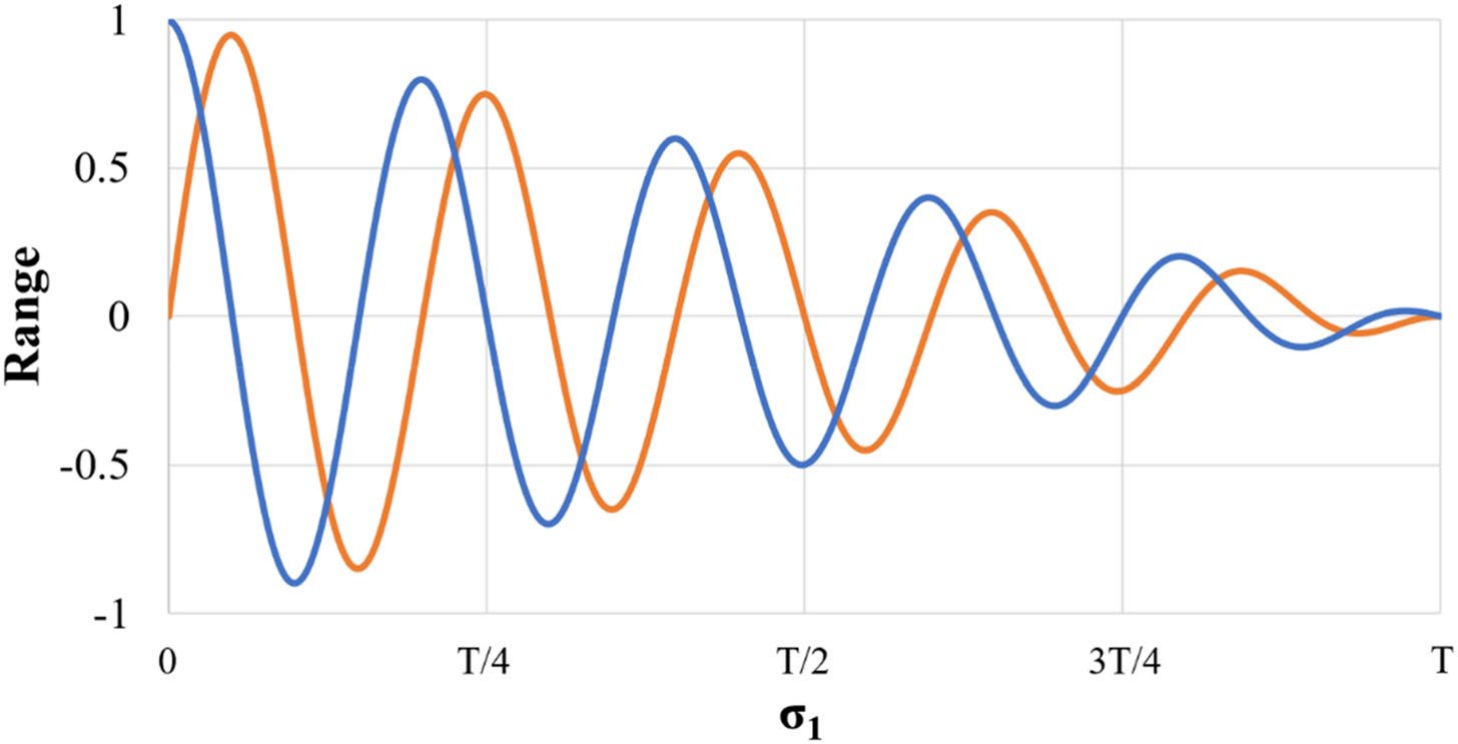
The range of sine and cosine exhibits a decreasing pattern.

Within each iteration, the range of the sine and cosine functions in Eq. (7) is dynamically adjusted to strike an optimal trade-off between exploitation and exploration (Fig. 4). This adaptive modification aims to efficiently identify fruitful spaces within the search area, ultimately enabling the attainment of the optimal solution. The adaptation process is governed by Eq. (8), where the constant value *v* is set to 2, *I*_*cur*_ represents the current iteration number, and *I*_*max*_ denotes the maximum iteration number.

8 $${\sigma }_{1}=v-{I}_{cur}\frac{v}{{I}_{max}}.$$

During the exploitation phase, as illustrated in the pseudocode of nSCA in Table 1, the solutions are updated using Eq. (7). Subsequently, a jumping condition, denoted as *JC* in Eq. (9), is employed to dynamically generate the opposite solution using Eq. (10). This procedure stands in contrast to the method adopted during the initial population generation phase. The objective equation evaluates both the original solution and its opposite, preserving the more optimal of the two and eliminating the less optimal. Such a practice guarantees the consistency of the population size, as articulated by Eq. (11).

9 $$JC=-{\left(\frac{{I}_{cur}}{{I}_{max}}\right)}^{2}+2\left(\frac{{I}_{cur}}{{I}_{max}}\right),$$

10 $$Create\, opposite \,solution\, {S}_{i}^{*}\,of\, {S}_{i}\, if\, {\sigma }_{6}\,<\,JC,$$

11 $${S}_{new}=\left\{\begin{array}{c}{S}_{i}\, if\, F\left({S}_{i}\right)\, is\, superior \,solution\\ {S}_{i}^{*} \,if\, F\,\left({S}_{i}^{*}\right)\, is\, superior\, solution\end{array}\right.,$$

where *S*_*i*_ represents the *i*th solution; *σ*_*6*_ is a uniformly distributed random variable between 0 and 1; ${S}_{i}^{*}$ represents the opposite solution of the *i*th solution created by OBL.

## Analysis of performance

In the realm of optimization, particularly in the exploration of evolutionary algorithms and metaheuristics, the efficacy of these algorithms is required to be validated to ensure their applicability in addressing real-world challenges. The performance of these optimization techniques is often benchmarked using specific test cases or established benchmark problems. Such benchmarks are provided to allow for a consistent platform, thereby facilitating an objective and uniform comparison across various algorithms. For the purposes of this research, the CEC 2019 and CEC 2021 test functions have been employed. These functions have been utilized to gauge the performance of nSCA in comparison to other well-established metaheuristic techniques.

### CEC 2019 test functions

The CEC 2019 dataset consists of ten complex composition functions within the suite^51^. For addressing large-scale optimization problems, these functions have been employed. The first three functions, F1 to F3, are characterized by various dimensions, as depicted in Table 2. In contrast, the functions F04 to F10 are set as 10-dimensional minimization challenges within the scope of [− 100, 100], and they have undergone shifts and rotations. Every function within the CEC 2019 is scalable, with the global optimum of these functions established at 1.

**Table 2:** CEC 2019 test functions.

Function	Name	n_dim_	Range	fmin
F1	Storn’s Chebyshev polynomial fitting problem	9	[− 8192, 8192]	1
F2	Inverse Hilbert matrix problem	16	[− 16,384, ,16384]	1
F3	Lennard–Jones minimum energy cluster problem	18	[− 4, 4]	1
F4	Shifted and rotated Rastrigin’s function	10	[− 100, 100]	1
F5	Shifted and rotated Griewank’s function	10	[− 100, 100]	1
F6	Shifted and rotated Weierstrass function	10	[− 100, 100]	1
F7	Shifted and rotated Schwefel’s function	10	[− 100, 100]	1
F8	Shifted and rotated expanded Schaffer’s F6 function	10	[− 100, 100]	1
F9	Shifted and rotated happy cat function	10	[− 100, 100]	1
F10	Shifted and rotated ackley function	10	[− 100, 100]	1

Results for the CEC 2019 test functions of nSCA, along with six other established metaheuristic algorithms (GA, PSO, MFO, ALO, MVO, and the original SCA), are provided in Tables 3 and 4. Each of the test functions was solved 30 times, with 50 search agents being utilized over 300 iterations. For the evaluation of nSCA’s performance, two essential statistical metrics, the average value (*avg*) and standard deviation (*std*), were determined.

**Table 3:** Statistical results obtained from different algorithms on CEC 2019 test functions.

Algorithm/function	Statistical metrics	*F1*	*F2*	*F3*	*F4*	*F5*
nSCA	*avg*	1.0000E+00	4.4360E+00	1.8642E+00	1.4672E+01	1.0438E+00
	*std*	5.3101E−10	4.1999E−02	2.5608E−01	2.3899E+00	1.4185E−02
GA	*avg*	3.2648E+05	4.5220E+02	7.6939E+00	1.1665E+02	1.4314E+00
	*std*	1.1632E+05	3.4891E+01	5.6725E−02	1.2830E+01	5.4219E−02
PSO	*avg*	1.3819E+07	9.0960E+02	1.0444E+01	2.4056E+02	1.9162E+00
	*std*	2.1059E+07	3.2540E+02	5.6372E−01	1.7613E+02	2.0756E−01
SCA	*avg*	2.2568E+05	2.6646E+02	5.1290E+00	1.2443E+02	1.5491E+00
	*std*	3.0806E+05	6.7367E+01	1.0833E+00	2.4558E+01	9.7463E−02
MFO	*avg*	3.3762E+08	2.1620E+03	1.0816E+01	8.6194E+03	3.9494E+00
	*std*	1.3608E+08	5.6290E+02	4.8941E−01	3.4470E+03	6.7227E−01
ALO	*avg*	2.5662E+06	4.2218E+02	5.0445E+00	3.0418E+01	1.3248E+00
	*std*	2.0764E+06	2.1465E+02	2.4198E+00	1.2777E+01	2.0214E−01
MVO	*avg*	1.6838E+06	2.6842E+02	7.5775E+00	2.9282E+01	1.1843E+00
	*std*	1.5459E+06	1.3917E+02	1.8665E+00	1.0332E+01	8.2334E−02

**Table 4:** Statistical results obtained from different algorithms on CEC 2019 test functions.

Algorithm/function	Statistical metrics	*F6*	*F7*	*F8*	*F9*	*F10*
nSCA	*avg*	9.3824E+00	1.0001E+00	1.0000E+00	1.2184E+00	1.9098E+01
	*std*	4.0091E−01	6.2581E−05	8.5380E−11	3.6162E−02	5.4587E+00
GA	*avg*	1.0651E+01	5.9839E+01	1.0119E+00	7.1370E+00	2.1416E+01
	*std*	1.4966E−01	5.8617E+01	4.6964E−03	4.3181E−01	1.7170E−02
PSO	*avg*	1.1121E+01	6.8933E+01	1.0008E+00	3.0083E+00	2.1452E+01
	*std*	5.1184E−01	8.7810E+01	9.1039E−04	3.2582E+00	2.6011E−01
SCA	*avg*	1.1069E+01	6.3272E+00	1.0020E+00	4.8350E+00	2.0601E+01
	*std*	5.8813E−01	1.7654E+00	8.6689E−04	8.1033E−01	2.5256E+00
MFO	*avg*	1.0302E+01	6.0386E+02	1.2980E+00	5.7099E+02	2.1465E+01
	*std*	1.2608E+00	1.4460E+02	1.1544E−01	2.0007E+02	1.1999E−01
ALO	*avg*	5.3504E+00	4.4431E+01	1.0000E+00	1.3973E+00	2.1038E+01
	*std*	1.4560E+00	1.8339E+02	2.4321E−11	2.2292E−01	8.2668E−02
MVO	*avg*	7.4261E+00	6.1456E+01	1.0000E+00	1.5303E+00	2.1103E+01
	*std*	1.2525E+00	2.0799E+02	8.7431E−07	1.6057E−01	4.3663E−02

In Tables 3 and 4, superiority in the majority of the CEC 2019 test cases was demonstrated by nSCA. An average value smaller than that of SCA, MFO, PSO, and GA in all 10 CEC 2019 test functions was attained by nSCA. In 9 of the CEC 2019 test functions, a smaller average value than that of MVO was achieved by nSCA. In 8 of the CEC 2019 test functions, a smaller average value than that of ALO was recorded by nSCA. The benefits of combining the RWS and OBL mechanisms were observed, as they aided in the initial exploration and contributed to the final convergence of the solutions identified early in the exploration phase.

### CEC 2021 test functions

For a comprehensive assessment, the effectiveness of nSCA was benchmarked against an array of state-of-the-art algorithms, utilizing the sophisticated functions delineated in the IEEE CEC 2021 test suite. The performance of nSCA was scrutinized based on shifted, rotated, and biased functions within this suite, spanning 10 dimensions, as detailed in Table 5. This methodology was employed to offer a deeper understanding of the capabilities of nSCA by setting it in direct comparison with other renowned algorithms such as GA, PSO, MFO, ALO, MVO, and the foundational SCA. In-depth insights into the IEEE CEC 2021 test suite can be found in Ref.^52^.

**Table 5:** CEC 2021 test functions.

Function	Name	ndim	Range	fmin
F1	Shifted and rotated Bent cigar function (F1 CEC-2017)	10	[− 100, 100]	100
F2	Shifted and rotated Schwefel’s function (F11 CEC-2014)	10	[− 100, 100]	1100
F3	Shifted and rotated Lunacek bi-Rastrigin function (F7 CEC-2017)	10	[− 100, 100]	700
F4	Expanded Rosenbrock’s plus Griewangk’s function (F15 CEC-2014)	10	[− 100, 100]	1900
F5	Hybrid function 1 (F17 CEC-2014)	10	[− 100, 100]	1700
F6	Hybrid function 2 (F15 CEC-2017)	10	[− 100, 100]	1600
F7	Hybrid function 3 (F21 CEC-2014)	10	[− 100, 100]	2100
F8	Composition function 1 (F21 CEC-2017)	10	[− 100, 100]	2200
F9	Composition function 2 (F23 CEC-2017)	10	[− 100, 100]	2400
F10	Composition function 3 (F24 CEC-2017)	10	[− 100, 100]	2500

Results of the CEC 2021 test functions for nSCA, alongside six other well-regarded metaheuristic algorithms (GA, PSO, MFO, ALO, MVO, and the original SCA), are elucidated in Tables 6 and 7. Following the approach used in the CEC 2019 test function assessment, each function underwent 30 trials, with the deployment of 50 search agents throughout 300 iterations. For the purpose of gauging the performance of nSCA, two statistical metrics, namely the average value (*avg*) and standard deviation (*std*), were extracted.

**Table 6:** Statistical results obtained from different algorithms on CEC 2021 test functions.

Algorithm/function	Statistical metrics	*F1*	*F2*	*F3*	*F4*	*F5*
nSCA	*avg*	2.6139E+06	9.7566E+04	2.2058E+04	1.9034E+03	6.1125E+03
	*std*	3.8102E+06	7.6190E+04	1.7970E+04	8.7061E−01	1.4966E+03
GA	*avg*	1.0005E+08	5.1855E+09	3.0612E+09	1.9675E+03	2.4600E+04
	*std*	1.7850E+08	1.3072E+10	1.0216E+10	7.3585E+01	9.1165E+03
PSO	*avg*	9.5844E+07	2.4241E+09	2.9732E+09	1.9891E+03	1.7675E+05
	*std*	2.6058E+08	4.3878E+09	6.7726E+09	2.3611E+02	1.5460E+05
SCA	*avg*	3.3244E+07	2.1955E+09	4.6439E+08	1.9043E+03	1.5350E+04
	*std*	1.5347E+07	9.4690E+08	2.1793E+08	6.9655E−01	7.1861E+03
MFO	*avg*	5.7981E+09	6.8269E+11	2.1017E+11	1.7674E+04	9.3913E+05
	*std*	2.2265E+09	3.3572E+11	8.4213E+10	2.7271E+04	7.9781E+05
ALO	*avg*	2.0900E+03	3.2377E+05	7.5700E+04	1.9021E+03	4.3610E+04
	*std*	2.2000E+03	3.8084E+05	7.5302E+04	1.1604E+00	3.9773E+04
MVO	*avg*	1.1209E+04	5.1402E+06	5.3232E+05	1.9022E+03	2.1333E+04
	*std*	8.7490E+03	9.8699E+05	5.2583E+05	6.4910E−01	6.1424E+03

**Table 7:** Statistical results obtained from different algorithms on CEC 2021 test functions (continued).

Algorithm/function	Statistical metrics	*F6*	*F7*	*F8*	*F9*	*F10*
nSCA	*avg*	2.1239E+03	6.3754E+03	2.3024E+03	2.5968E+03	3.0088E+03
	*std*	2.9213E+02	2.2236E+03	9.3241E−01	1.7917E+01	1.8385E+01
GA	*avg*	4.8594E+03	7.1530E+03	2.3345E+03	3.1832E+03	3.1595E+03
	*std*	3.7275E+03	5.2281E+03	1.1242E+01	8.3419E+02	4.1794E+01
PSO	*avg*	7.2962E+03	1.5042E+05	2.3130E+03	3.0133E+03	3.0895E+03
	*std*	5.0285E+03	5.4994E+05	4.4205E+00	3.3501E+02	6.3185E+01
SCA	*avg*	2.9617E+03	1.1761E+04	2.3087E+03	2.8274E+03	3.0072E+03
	*std*	1.0790E+03	4.5072E+03	1.1630E+00	8.4929E+01	1.1181E+01
MFO	*avg*	2.9666E+04	7.2657E+06	2.3457E+03	5.5363E+03	3.4342E+03
	*std*	1.7217E+04	6.1941E+06	9.3688E+00	6.0756E+02	2.1886E+02
ALO	*avg*	6.2181E+03	2.5595E+04	2.3075E+03	2.5974E+03	3.0110E+03
	*std*	5.7222E+03	2.6519E+04	2.9237E+00	3.3996E+01	3.7818E+01
MVO	*avg*	2.4545E+03	2.3408E+03	2.3036E+03	2.6071E+03	2.9798E+03
	*std*	2.1974E+03	2.3907E+02	1.8328E+00	5.2987E+01	2.5083E+01

Tables 6 and 7 reveal the dominance of nSCA across a significant portion of the CEC 2021 test cases. Specifically, nSCA secured an average value lower than those of MFO, PSO, and GA across all 10 CEC 2021 test functions. Moreover, in 9 out of these 10 functions, nSCA surpassed the original SCA. In a comparison with ALO, nSCA managed to record a lower average in 8 functions, whereas MVO achieved this distinction in 6 functions.

**Figure 5:**
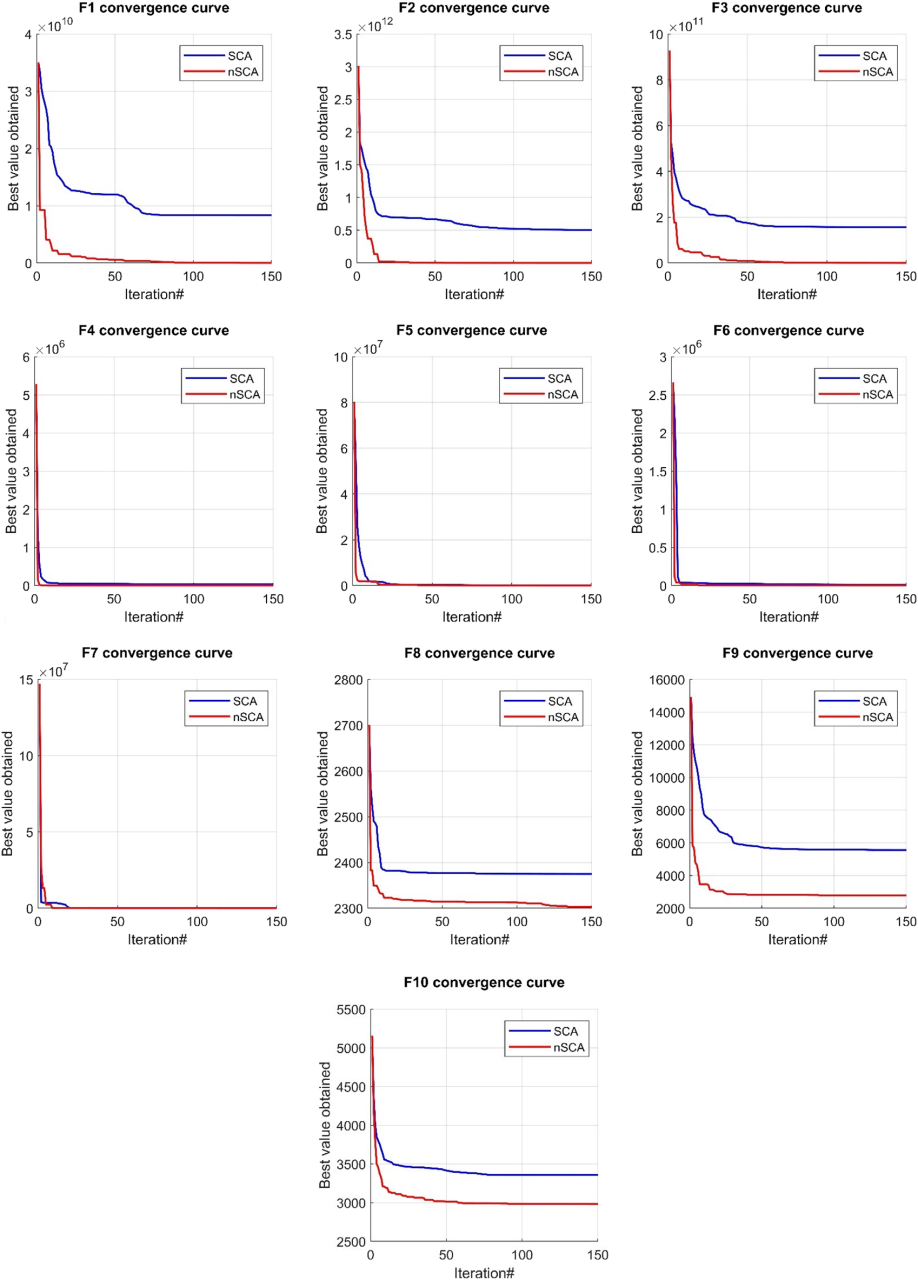
Convergence behavior of nSCA and SCA on CEC 2021 test functions.

Figure 5 offers a visual depiction of the convergence trajectories of both nSCA and its original version, SCA, across the CEC 2021 test functions. From Fig. 5, it is clear that nSCA significantly excels over the original SCA in terms of identifying superior solutions. Additionally, as evidenced in Fig. 5, the effectiveness of nSCA in locating the global optimal solution and avoiding local optima is attributed to its integration of the RWS and OBL mechanisms. These mechanisms not only enable nSCA to induce sudden shifts in solution vectors but also, through the juxtaposition of fitness values between original and OBL-generated solutions, facilitate the retention of more favorable options. This capability equips nSCA to discern promising zones within the search landscape, ensuring a thorough exploration and the subsequent identification of optimal outcomes.

## Practical application of nSCA

The primary objective of this section is to evaluate the effectiveness of nSCA in addressing a range of practical technical optimization challenges characterized by multiple inequality constraints. The emphasis lies in understanding the ability of nSCA to adeptly manage these constraints during the optimization procedure.

### Cantilever beam design problem

**Figure 6:**
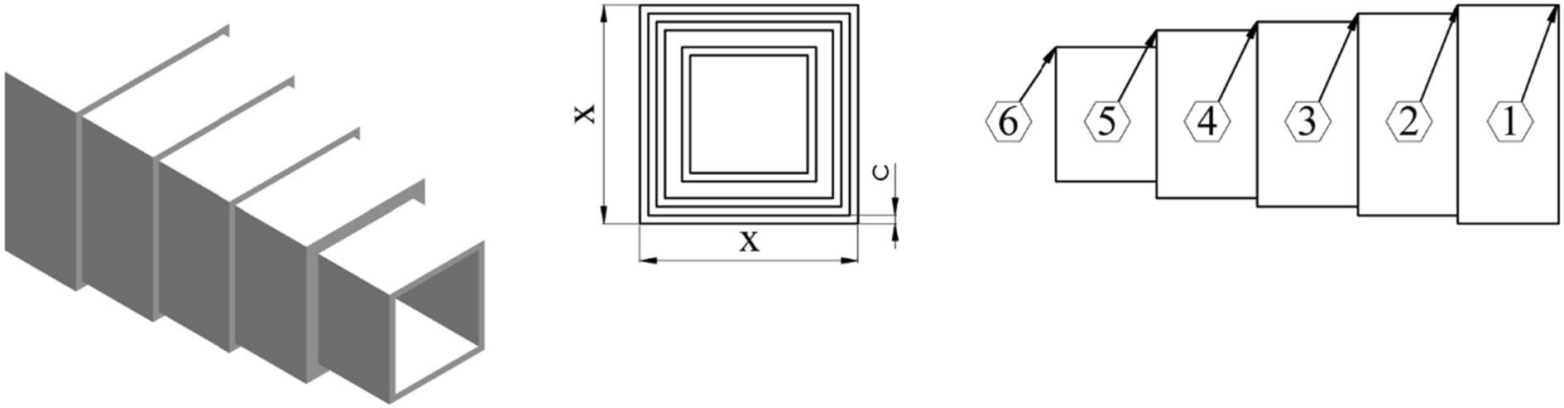
Cantilever beam design problem.

Figure 6 presents a visual depiction of five parameters that define the cross-sectional geometry of cubes within the beam. This particular beam is assembled from five distinct square blocks. While the foremost block remains fixed, the fifth one is subjected to a vertical load. The central objective of this optimization task is to minimize the weight of a cantilever beam composed of hollow square blocks. Subsequent equations elaborate on the mathematical underpinnings that frame this complex challenge. Within the nSCA framework, any solution that fails to satisfy the constraints is penalized by assigning it an exceptionally large fitness value. The incorporation of OBL and RWL techniques, which facilitate abrupt adjustments to the non-conforming solution, primes the algorithm to generate an improved, compliant solution from its predecessor. Such methodologies empower the nSCA to channel its search in the direction of solutions that comply with the established constraints.

Consider:

12 $$\overrightarrow{x}=\left[{x}_{1}{ x}_{2} {x}_{3} {x}_{4} {x}_{5}\right].$$

Minimize:

13 $$f\left(\overrightarrow{x}\right)=0.6224\left({x}_{1}+{x}_{2}+{x}_{3}+{x}_{4}+{x}_{5}\right).$$

Subject to:

14 $$g\left(\overrightarrow{x}\right)=\frac{61}{{x}_{1}^{3}}+\frac{27}{{x}_{2}^{3}}+\frac{19}{{x}_{3}^{3}}+\frac{7}{{x}_{4}^{3}}+\frac{1}{{x}_{5}^{3}}-1\le 0.$$

Variable range:

15 $$0.01\le {x}_{1}, {x}_{2}, {x}_{3}, {x}_{4}, {x}_{5}\le 100.$$

Table 8 provides a detailed evaluation of the results pertaining to the problem. Evidently, nSCA consistently produces solutions that either match or surpass the performance of advanced optimization techniques such as SCSO, PSO^53^, RCGO^54^, ERHHO^55^, GSA^56^, GCA_I^57^, GCA_II^57^ and MMA^57^. This observation underscores the formidable capability of the algorithm in adeptly addressing and optimizing complex constrained challenges. Furthermore, these outcomes highlight the practical utility of nSCA in sectors like engineering and related fields, emphasizing its competence in navigating challenging problem landscapes.

**Table 8:** The best design obtained by different algorithms on cantilever beam design problem.

Optimization technique	Optimal parameters					Optimal weight
	*x*_1_	*x*_2_	*x*_3_	*x*_4_	*x*_5_	
nSCA (This study)	5.944606	4.865280	4.503500	3.492579	2.134620	1.303342
SCSO^53^	6.0164	5.3060	4.4935	3.5059	2.1516	1.339952
PSO^53^	6.0040	5.2950	4.4915	3.5125	2.1710	1.339983
RCGO^54^	6.020877	6.020877	6.020877	6.020877	6.020877	1.33996
ERHHO^55^	6.0509	5.2639	4.514	3.4605	2.1878	1.3402
GSA^56^	5.6052	4.9553	5.6619	3.1959	3.2026	1.41
GCA_I^57^	6.0100	5.3000	4.4900	3.4900	2.1500	1.3400
GCA_II^57^	6.0100	5.3000	4.4900	3.4900	2.1500	1.3400
MMA^57^	6.0100	5.3000	4.4900	3.4900	2.1500	1.3400

### Truss structure design problem using continuous variables

Truss optimization represents a complex facet of structural engineering and design, focused primarily on discerning the most resource-efficient configurations for truss structures. Defined as skeletal assemblies composed of straight members intersecting at joints, trusses are foundational in both architectural and civil engineering realms. They play a crucial role in supporting various types of loads, especially in the contexts of buildings, bridges, and other diverse structural entities.

The primary goal of truss optimization is to conceive a truss design capable of bearing the stipulated loads with the least material consumption. This efficiency is attained by fine-tuning the section of each truss member. The aim is to ensure that every member endures minimal stress while abiding by specific design constraints. Within truss optimization, the design variables encompass the cross-sectional areas of the truss members, the precise locations of the joints, and a myriad of geometric parameters delineating the truss’s shape and configuration. The mathematical representations pertinent to this optimization challenge can be delineated as follows:

Consider:

16 $$\overrightarrow{x}=\left[{x}_{1}{ x}_{2}\dots {x}_{N}\right].$$

Objective function:

17 $$f\left(\overrightarrow{x}\right)=W=\sum_{i=1}^{N}{\gamma }_{i}{L}_{i}{A}_{i}.$$

Subject to:

18 $${\sigma }_{\text{min}}\le {\sigma }_{i}\le {\sigma }_{\text{max}},$$

19 $${\delta }_{\text{min}}\le {\delta }_{j}\le {\delta }_{\text{max}},$$

20 $${A}_{\text{min}}\le {A}_{i}\le {A}_{\text{max}}.$$

In Eq. (17), *W* denotes the total weight of the truss structure. *γ*_*i*_ represents the material density of the *i*th truss member, while *A*_*i*_ and *L*_*i*_ signify the cross-sectional area and length of the *i*th member, respectively. *N* stands for the total number of members in the truss structure. Equation (18) is imperative for ensuring that the truss design complies with the stress constraints. Within this equation, *σ*_*i*_ refers to the stress experienced by the *i*th member. *σ*_min_ and *σ*_max_ are, respectively, the minimum and maximum permissible stresses. Equation (19) is formulated to ascertain that the truss design adheres to the deflection constraints. In this context, *δ*_*j*_ is the deflection at the *j*th node, and *δ*_min_ and *δ*_max_ correspond to the minimum and maximum allowable deflections, respectively. Lastly, Eq. (20) ensures that the truss design remains within the geometric constraints. For this equation, *A*_*i*_ is the cross-sectional area of the *i*th member, and *A*_min_ and *A*_max_ represent the smallest and largest permissible cross-sectional areas for the truss members, respectively.

To provide an unbiased comparison among truss design problems, the necessity of multiple independent evaluations was emphasized. Consequently, ten independent runs were undertaken. In each instance, a group of 50 search agents was utilized, with each agent progressing through 250 iterations.

#### 10-Bar truss structure design problem

**Figure 7:**
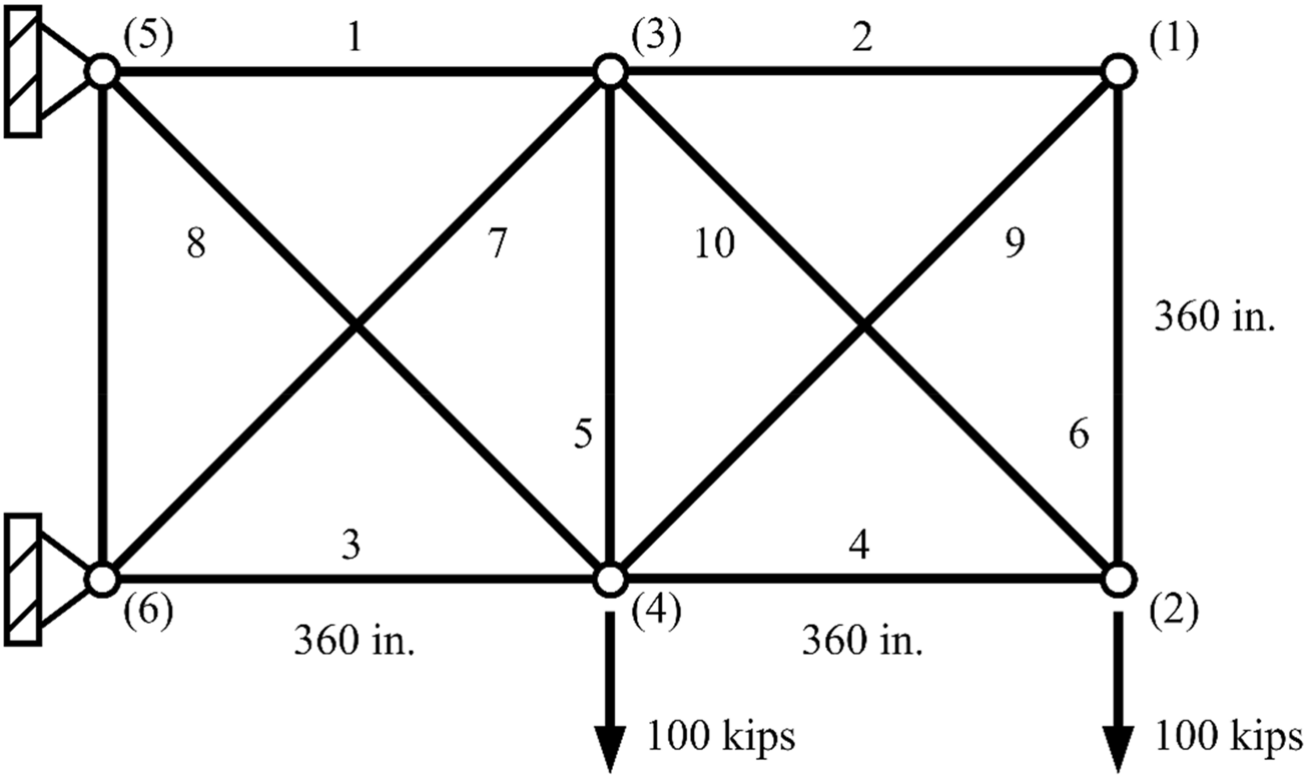
10-Bar truss structure problem.

In order to benchmark the performance of the nSCA in comparison with other optimization techniques tailored for continuous variables, an augmented 10-bar truss design is introduced, as illustrated in Fig. 7. This design specification permits the truss’s cross-sectional areas to vary between a range of 0.1 in^2^ and 35.0 in^2^.

The material selected for this truss possesses distinctive characteristics. Specifically, it carries a unit weight of 0.1 lb/in^3^ and is characterized by a modulus of elasticity set at 10^7^ psi.

The design of the truss is governed by certain predefined conditions:

- The stress magnitudes in any given truss member must not surpass an acceptable range of ± 25 ksi.
- All nodal deflections, be they vertical or horizontal, must be confined within a limit of ± 2.0 in.

These predetermined conditions aim to guarantee the truss’s peak performance, ensuring it is consistent with its established design criteria and operational requirements.

Table 9 presents a comparative analysis of the optimal solution obtained using nSCA alongside results from various other optimization techniques. It is significant to highlight that the results derived from PSO^62^ and HS^61^ are characterized by constraint violations. When utilizing nSCA, the design records a weight of 5061.0548 lb after 10,950 equation evaluations and presents a standard deviation (SD) of 0.5491. Such performance is superior to that of both EHS^59^ and SAHS^59^. Although the design from ABC-AP^60^ has a weight of 5060.88 lb and emerges as the most optimal on the scale, it necessitates an extensive 500,000 function evaluations. Conversely, the design from TLBO^58^ with a weight of 5060.973 lb, aligns closely with the outcome from ABC-AP^60^ but necessitates 13,767 function evaluations. From the data collated in Table 9, nSCA not only produces a solution comparable to those of other renowned algorithms but also excels in terms of computational efficiency.

**Table 9:** The best design obtained by different algorithms on the 10-bar truss structure using continuous variables.

Variables (mm^2^)	nSCA	TLBO ^58^	SAHS ^59^	EHS ^59^	ABC-AP ^60^	HS ^61^	PSO ^62^
1	30.265338	30.6684	30.394	30.208	30.548	30.150	29.999
2	0.100000	0.1000	0.100	0.100	0.100	0.102	0.100
3	23.301629	23.1584	23.098	22.698	23.180	22.710	23.268
4	15.361201	15.2226	15.491	15.275	15.218	15.270	15.129
5	0.100000	0.1000	0.100	0.100	0.100	0.102	0.100
6	0.561455	0.5421	0.529	0.529	0.5510	0.544	0.554
7	20.947396	21.0255	21.189	7.558	21.058	21.560	21.232
8	7.426226	7.4654	7.488	21.559	7.4630	7.541	7.454
9	0.100000	0.1000	0.100	0.100	0.100	0.100	0.100
10	21.656878	21.4660	21.342	21.491	21.501	21.450	21.670
Optimal weight (lb)	5061.0548	5060.973	5061.42	5062.39	5060.88	5057.88	5059.85
Mean weight (lb)	5061.8713	5064.808	5061.95	5063.73	N/A	N/A	5067.51
SD	0.5491	6.3707	0.71	1.98	N/A	N/A	17.509
NFE_max_	12,500	N/A	30,000	30,000	N/A	N/A	N/A
Best NFE	10,950	13,767	7081	9791	500,000	20,000	10,194
Constraint tolerance (%)	None	None	None	None	None	0.191	0.109

#### 25-Bar truss structure design problem

**Figure 8:**
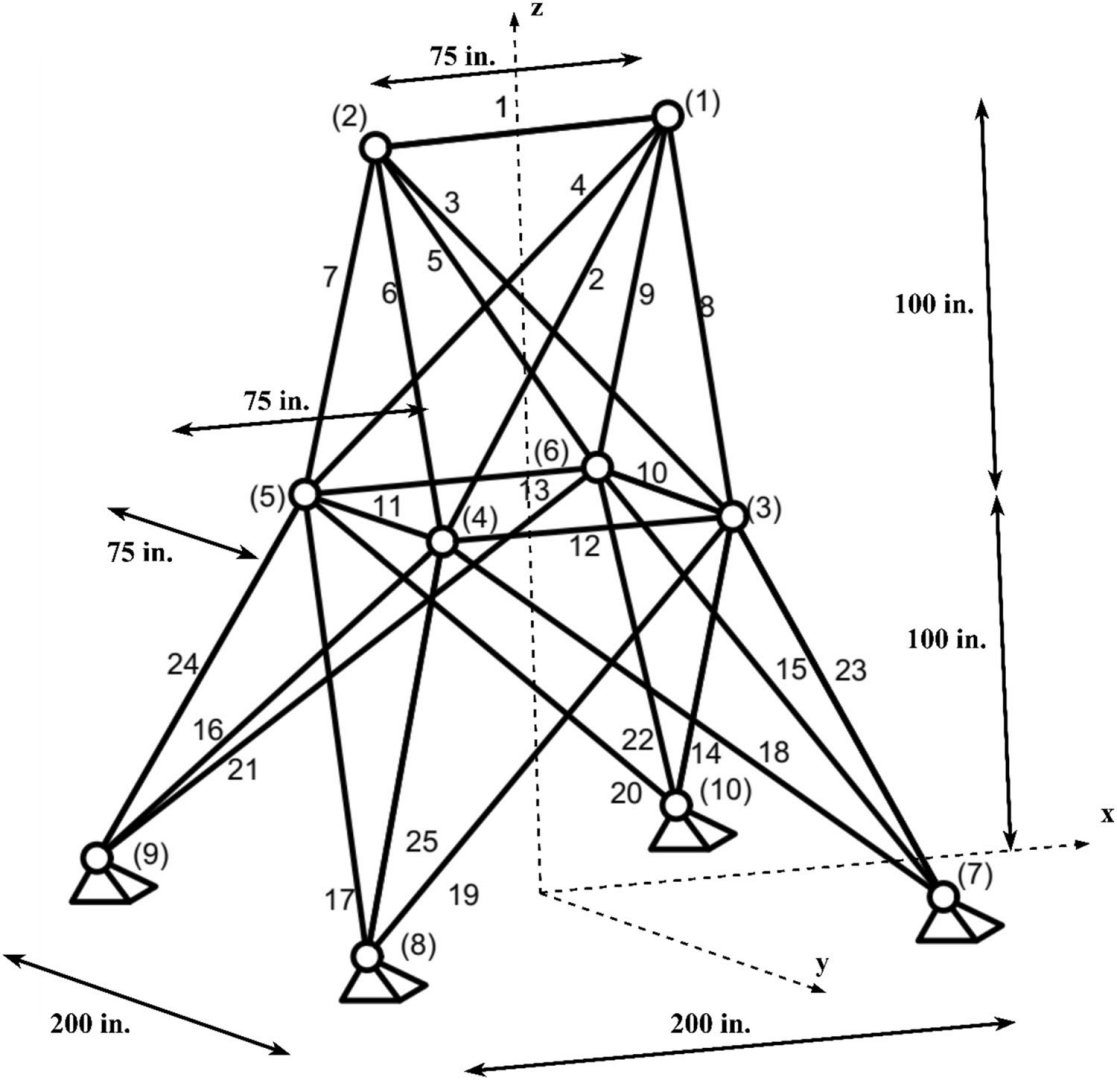
25-bar truss structure problem.

The 25-bar truss problem, as illustrated in Fig. 8, encompasses two distinct load scenarios, detailed in Table 10. This truss structure is divided into eight symmetrical segments, with each segment subject to its own stress limitations as outlined in Table 11.

**Table 10:** Multiple loading cases for 25-bar truss structure using continuous variables.

Case	Note	P_x_ (kips)	P_y_ (kips)	P_y_ (kips)
1	1	1.0	10.0	− 5.0
	2	0.0	10.0	− 5.0
	3	0.5	0.0	0.0
	6	0.5	0.0	0.0
2	1	0.0	20.0	− 5.0
	2	0.0	− 20.0	− 5.0

**Table 11:** Element groups and corresponding allowable stresses for 25-bar truss structure using continuous variables.

Group	Elements	Compression (ksi)	Tension (ksi)
1	A1	35.092	35.0
2	A2–A5	11.590	35.0
3	A6–A9	17.305	35.0
4	A10, A11	35.092	35.0
5	A12, A13	35.092	35.0
6	A14–A17	6.759	35.0
7	A18–A21	6.959	35.0
8	A22–A25	11.082	35.0

The selected material for the truss structure possesses the following attributes:

- A density of 0.1 lb/in^3^.
- A modulus of elasticity of 10,000 ksi.

Constraints on nodal movements have been enforced, ensuring displacements do not exceed ± 0.35 inches in any x, y, or z direction. These constraints are grounded in the findings presented in Ref.^63^.

In this problem, the design variables are continuous. Moreover, the bars within the truss can have cross-sectional areas ranging from a minimum of 0.01 in^2^ to a maximum of 3.40 in^2^. This range facilitates the optimization of the truss within the prescribed constraints and stipulations.

Table 12 offers a comprehensive comparison between the designs produced using nSCA and those generated by other optimization techniques. Notably, the most efficient 25-bar truss configuration realized by nSCA weighs 545.1630 lb, ascertained following 10,350 evaluation equations, accompanied by a standard deviation (SD) of 0.1820. From the information provided in Table 12, it becomes evident that nSCA surpasses several other techniques, including PSO^64^, MSPSO^64^, HSPSO^65^, IRO^66^, TLBO^58^, ACO ^67^ and STA^68^. This assessment underscores the capability of nSCA in achieving superior performance and computational efficiency, particularly in the context of the 25-bar truss problem with continuous variables, thereby solidifying its edge over other metaheuristic methodologies.

**Table 12:** The best design obtained by different algorithms on the 25-bar truss structure using continuous variables.

Variables (in^2^)	nSCA (This study)	STA ^68^	TLBO ^58^	IRO ^66^	MSPSO ^64^	PSO ^64^	HSPSO ^65^	ACO ^67^
Group 1	0.010003	0.0102	0.0100	0.0112	0.01	0.01	0.010	0.01
Group 2	1.985021	1.9866	1.9878	1.9766	1.9848	1.9503	1.970	2.0000
Group 3	2.996736	2.9943	2.9914	3.0099	2.9956	3.0408	3.016	2.966
Group 4	0.010000	0.01	0.0102	0.01	0.01	0.01	0.01	0.01
Group 5	0.010001	0.01	0.0100	0.01	0.01	0.01	0.01	0.012
Group 6	0.683104	0.6835	0.6825	0.6842	0.6852	0.6929	0.694	0.689
Group 7	1.677167	1.677	1.6775	1.6783	1.6778	1.6866	1.681	1.679
Group 8	2.662307	2.6626	2.6640	2.6571	2.6599	2.6362	2.643	2.668
Optimal weight (lb)	545.1630	545.175	545.175	545.19	545.172	545.22	545.19	545.53
Mean weight (lb)	545.3234	552.43	545.483	545.35	546.03	549.96	N/A	546.34
SD	0.1820	14.08	0.306	N/A	0.8	9.91	N/A	0.94
NFE_max_	12,500	12,000	N/A	15,000	25,000	25,000	150,000	16,500
Best NFE	10,350	11,985	12,199	12,200	10,800	18,400	12,500	4700

### Capacity vehicle routing problem

The capacitated vehicle routing problem (CVRP), inherently discrete in nature, holds a foundational topic within the realms of operations research and logistics, as evidenced by a comprehensive body of research^69^. Essentially, the CVRP seeks to identify the most efficient strategy for distributing goods from a singular depot to a predetermined set of customers. This task is accomplished by deploying a fleet of vehicles, each of which returns to the depot after its delivery. This concept can be succinctly captured in a mathematical formulation:

Consider:

$$D=total\, distance\, travelled\, by\, all \,units,$$

$${x}_{ijt}=\left\{\begin{array}{l}1, vehicle \,t\, depart\, from\, i\, to\, j\\ 0, otherwise\end{array}\right.;{y}_{it}=\left\{\begin{array}{c}1, customer \,i \,is\, served\, by unit t\\ 0, otherwise\end{array}\right..$$

Objective function:

21 $$Min D=\sum_{i=0}^{k}\sum_{j=0}^{k}\sum_{t=1}^{h}{c}_{ij}{x}_{ijt},$$

where *x*_*ijt*_ is a binary variable that indicates the selection of a route. Specifically, *x*_*ijt*_ is set to 1 if the route between customer *i* and customer *j* is chosen by the *t*th vehicle, and 0 otherwise. *c*_*ij*_ denotes the cost associated with traveling from customer *i* to customer *j*.

As outlined by Shan and Wang^70^, the CVRP operates under two principal constraints:

- *Single visit requirement* Each customer must be serviced exactly once, ensuring not just efficiency, but also punctuality in deliveries.
  22 $$\sum_{i=0}^{k}{x}_{ijt}={y}_{jt};j=\mathrm{1,2},\dots ,k;t=\mathrm{1,2},\dots ,h,$$
  23 $$\sum_{i=0}^{k}{x}_{ijt}={y}_{it};j=\mathrm{1,2},\dots ,k;t=\mathrm{1,2},\dots ,h,$$
  24 $$\sum_{t=1}^{h}{y}_{it}=\left\{\begin{array}{c}1; i=\mathrm{1,2},3,\dots ,k\\ h;i=0\end{array}\right\}.$$
  Equations (22) and (23) collaboratively ensure that each vehicle follows a unique route to serve every customer. Specifically, Eq. (22) mandates that each customer is visited only once, while Eq. (23) dictates that every vehicle must cater to at least one customer. In the context of Eq. (24), it is stipulated that a given customer can only be served by a single vehicle. However, an exception is carved out for the central depot or warehouse, which may be accessed by *h* vehicles, signifying the total fleet allocated for the operation.
- *Vehicle capacity constraint* Every vehicle within the fleet possesses a predetermined carrying capacity. As a result, the cumulative volume or weight of goods allocated to a particular route must not surpass this stipulated capacity.
  25 $$\sum_{i=0}^{k}{g}_{i}{y}_{it}\le {q}_{t}{y}_{it};t=\mathrm{1,2},\dots ,h.$$
  Equation (25) enforces a restriction on the carrying capacity of each vehicle, ensuring that it does not surpass its predetermined volume or weight during any given trip. Within this equation, *g*_*i*_ represents the demand of the *i*th client, with *i* varying from 1 through *k*—the total number of clients. The term *h* symbolizes the entire count of vehicles engaged in the operation. Concurrently, *q*_*t*_ designates the capacity of the *t*th vehicles, with *t* ranging from 1 to *h*.

#### 8-Customer problems

In this problem, there exists a central warehouse serving eight distinct customers. Two trucks, each boasting a carrying capacity of eight units, are deployed for this operation. Relevant data encompassing the distance matrix and individual customer demands are detailed in Table 13. The primary objective centers around optimizing the delivery routes for these trucks, aiming to minimize the total distance covered while concurrently respecting the inherent constraints of the VRP. To ensure robustness and maintain consistency in results, every algorithm was run 20 times, employing 20 search agents, and was subjected to a total of 50 iterations.

**Table 13:** Distance matrix and delivery requirements of 8-customer problem ^71^.

Node	0	1	2	3	4	5	6	7	8	Demand
0	0	4	6	7.5	9	20	10	16	8	
1	4	0	6.5	4	10	5	7.5	11	10	1
2	6	6.5	0	7.5	10	10	7.5	7.5	7.5	2
3	7.5	4	7.5	0	10	5	9	9	15	1
4	9	10	10	10	0	10	7.5	7.5	10	2
5	20	5	10	5	10	0	7	9	7.5	1
6	10	7.5	7.5	9	7.5	7	0	7	10	4
7	16	11	7.5	6	7.5	9	7	0	10	2
8	8	10	7.5	15	10	7.5	10	10	0	2

Table 14 presents a comparative analysis of results derived from various algorithms. Among them, nSCA distinguished itself by exhibiting exceptional efficiency. This superior performance becomes evident when considering its average percentage deviation (APD) from the optimal result. The most favorable solution identified for this specific problem had a total travel distance of 67.5 units. With an APD value of 0.33%, nSCA surpassed the performances of the original SCA^36^, DA^36^, ALO^36^, PSO^36^, MHPSO^71^, DPGA^71^, and SGA^71^. Their corresponding APD values were recorded as 0.78%, 1.81%, 2.44%, 2.15%, 2.04%, 3.04%, and 4.33%, respectively. Such data distinctly emphasize the robustness of nSCA, especially in addressing discrete problem-solving challenges.

**Table 14:** Results of different algorithms on 8-customer problem.

Algorithm	Solution set obtained by algorithm					Max	Min	Mean	Standard deviation	APD (%)
nSCA (This study)	67.5	68	67.5	67.5	67.5	68.5	67.5	67.725	0.33	0.33
	68.5	67.5	67.5	67.5	67.5					
	68.5	68	68	68	68					
	67.5	67.5	67.5	67.5	67.5					
SCA^36^	69	68	69	68	68	69.5	67.5	68.025	0.60	0.78
	68	69.5	67.5	67.5	68					
	69	68	67.5	67.5	67.5					
	68	67.5	68	67.5	67.5					
DA^36^	71.5	67.5	71.5	68	67.5	71.5	67.5	68.725	1.30	1.81
	69	70	70.5	68	69					
	70	67.5	67.5	69	68					
	67.5	68	68	67.5	69					
ALO^36^	71.5	68	71.5	68	67.5	71.5	67.5	69.15	1.38	2.44
	69	70	70.5	68	69					
	70	68	71	69	68					
	71.5	68	68	67.5	69					
PSO^36^	67.5	70	70	69	69	70	67.5	68.95	0.89	2.15
	68	69	70	70	68.5					
	68.5	68.5	67.5	68	70					
	70	67.5	69.5	69	69.5					
MHPSO^71^	69.5	67.5	69	69	70	70	67.5	68.875	0.97	2.04
	69.5	70	69	67.5	67.5					
	69	69.5	69	70	67.5					
	70	69	67.5	70	67.5					
DPGA^71^	70	69	67.5	71	69	72	67.5	69.55	1.36	3.04
	70.5	72	67.5	71.5	69					
	67.5	69	71	70	67.5					
	70.5	69	69.5	71	69					
SGA^71^	69	72	73.5	69	70	75.5	67.5	70.425	2.03	4.33
	71	67.5	69	69	75.5					
	70	69.5	69	73	69					
	74	70	69.5	69	70					

**Figure 9:**
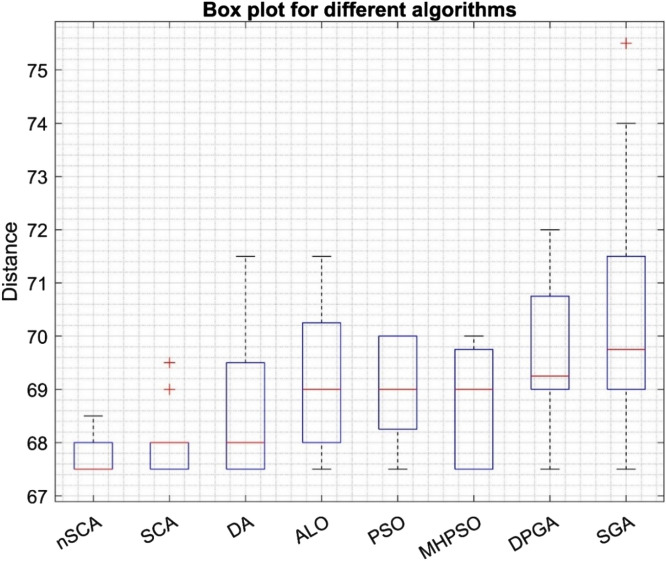
Boxplot of nSCA, SCA, DA, ALO, PSO, MHPSO, DPGA, and SGA on 8-customer problem.

**Figure 10:**
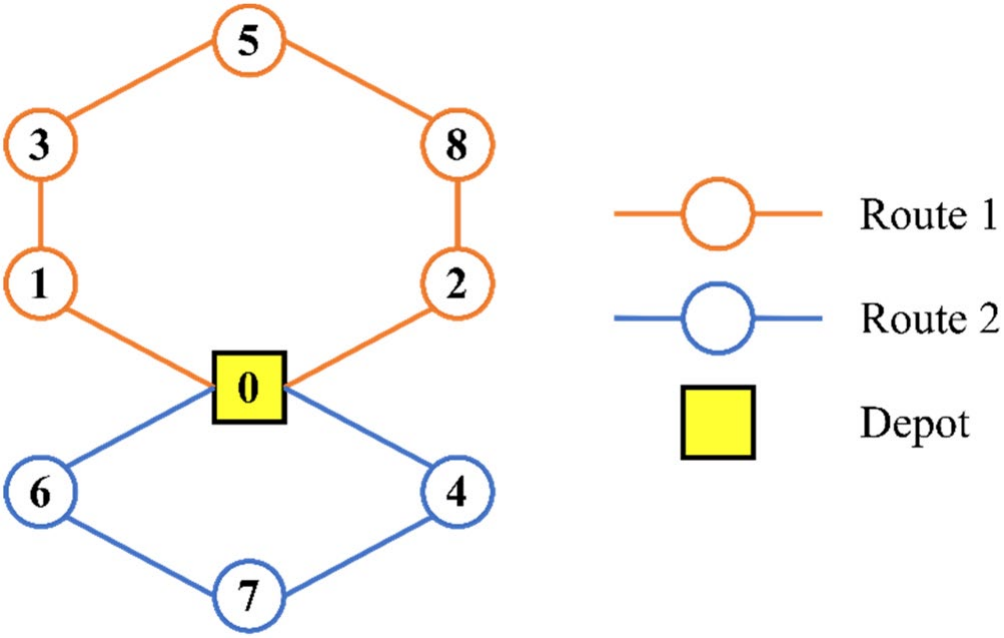
Best solution of the 8-customer problem.

Figure 9 features a boxplot that contrasts nSCA with other metaheuristic methods, accentuating the efficacy of nSCA. The detailed routing for the two vehicles, representing the optimal solution, can be found in Table 15. For enhanced visual comprehension, this routing is also depicted graphically in Fig. 10.

**Table 15:** Routing of vehicles and distance obtained by nSCA on 8-customer problem.

Routes of the vehicles on 8-customer problem		Distance
Route 1.	0 → Customer 1 → Customer 3 → Customer 5 → Customer 8 → Customer 2 → 0	34
Route 2.	0 → Customer 6 → Customer 7 → Customer 4 → 0	33.5
Total distance: 67.5 units

#### Real CVRP in Vietnam: 16-customer problems

In a practical application of the CVRP, delivery data from a delivery company in Vietnam was analyzed. Serving 16 customers through a hub-and-spoke distribution model, the supplier operates a fleet of three delivery vehicles, each with a carrying capacity of 70 units. Distinguished from the traditional TSP, this case introduces added complexity due to the presence of multiple vehicles and capacity constraints. Distance and demand data for the 16 customers were processed and converted into a distance matrix, as detailed in Table 16.

**Table 16:** Distance matrix and delivery requirements of 16-customer problem.

Node	0	1	2	3	4	5	6	7	8	9	10	11	12	13	14	15	16	Demand
0	0	67	54	33	15	50	51	46	40	12	48	61	25	52	42	43	45	0
1	67	0	121	35	64	116	96	74	48	58	50	85	91	70	31	110	55	13
2	54	121	0	87	60	17	61	77	88	64	93	86	31	86	94	13	88	4
3	33	35	87	0	35	81	65	47	34	23	30	61	57	47	11	76	33	5
4	15	64	60	35	0	60	66	60	28	22	57	75	35	65	45	52	56	10
5	50	116	17	81	60	0	45	63	89	58	82	70	26	72	87	11	76	3
6	51	96	61	65	66	45	0	25	89	49	49	26	42	33	65	49	43	12
7	46	74	77	47	60	63	25	0	76	38	25	16	50	9	43	64	19	15
8	40	48	88	34	28	89	89	76	0	40	64	92	63	79	44	80	66	17
9	12	58	64	23	22	58	49	38	40	0	36	53	34	43	30	53	34	6
10	48	50	93	30	57	82	49	25	64	36	0	35	62	20	22	80	6	12
11	61	85	86	61	75	70	26	16	92	53	35	0	62	15	56	73	30	7
12	25	91	31	57	35	26	42	50	63	34	62	62	0	59	63	19	58	13
13	52	70	86	47	65	72	33	9	79	43	20	15	59	0	41	73	15	8
14	42	31	94	11	45	87	65	43	44	30	22	56	63	41	0	82	26	9
15	43	110	13	76	52	11	49	64	80	53	80	73	19	73	82	0	75	10
16	45	55	88	33	56	76	43	19	66	34	6	30	58	15	26	75	0	14

The core objective of this study revolves around optimizing the delivery process for a set of 16 customers using a fleet comprising three vehicles. The goal is to curtail the overall distance covered while staying within the boundaries of the CVRP constraints. This challenge was tackled using nSCA, alongside other esteemed algorithms like GA, PSO, MFO, ALO, MVO, and the original SCA. To ensure a balanced comparison, each algorithm underwent 20 runs, deploying 50 search agents, and spanning 400 iterations for all CVRP test instances. The outcomes generated by nSCA and the other methodologies are detailed in Table17.

**Table 17:** Results of different algorithms on 16-customer problem.

Algorithm	Solution set obtained by algorithm					Max	Min	Mean	Standard deviation	APD (%)
nSCA	553	568	473	514	463	568	463	503.75	31.5	8.80
	488	528	468	519	479					
	492	515	465	537	489					
	486	510	463	554	511					
SCA	595	518	571	528	582	665	518	584.7	40.9	26.29
	614	665	561	620	588					
	594	658	542	616	546					
	575	647	540	565	569					
GA	719	712	569	622	632	730	569	649.1	46.2	40.19
	730	692	629	625	597					
	706	649	677	626	600					
	632	670	641	680	574					
PSO	612	620	658	570	599	658	552	599.2	26.3	29.42
	605	605	568	560	597					
	610	628	622	552	593					
	600	638	569	593	585					
ALO	706	731	706	605	622	731	605	675.1	32.1	45.81
	662	727	689	665	666					
	640	636	649	672	700					
	689	680	687	685	685					
MFO	518	550	570	589	693	699	518	595.6	50.0	28.64
	532	591	579	575	658					
	538	594	699	611	570					
	544	622	593	655	631					
MVO	552	644	582	676	625	687	551	617.9	42.9	33.46
	560	551	687	632	617					
	576	655	686	631	609					
	584	586	604	680	621					

As evident from Table 17, nSCA clearly stands out in terms of efficiency. The optimal solution for this problem, identified using nSCA, corresponded to a total travel distance of 463 units. nSCA showcased an APD value of 8.80%, which was superior to the performances of the original SCA, GA, PSO, ALO, MFO, and MVO. The APD values for these methods were observed to be 26.29%, 40.19%, 29.42%, 45.81%, 28.64%, and 33.46%, respectively. This information underscores the strengths of nSCA, particularly when confronted with large-scale discrete problem-solving scenarios.

**Figure 11:**
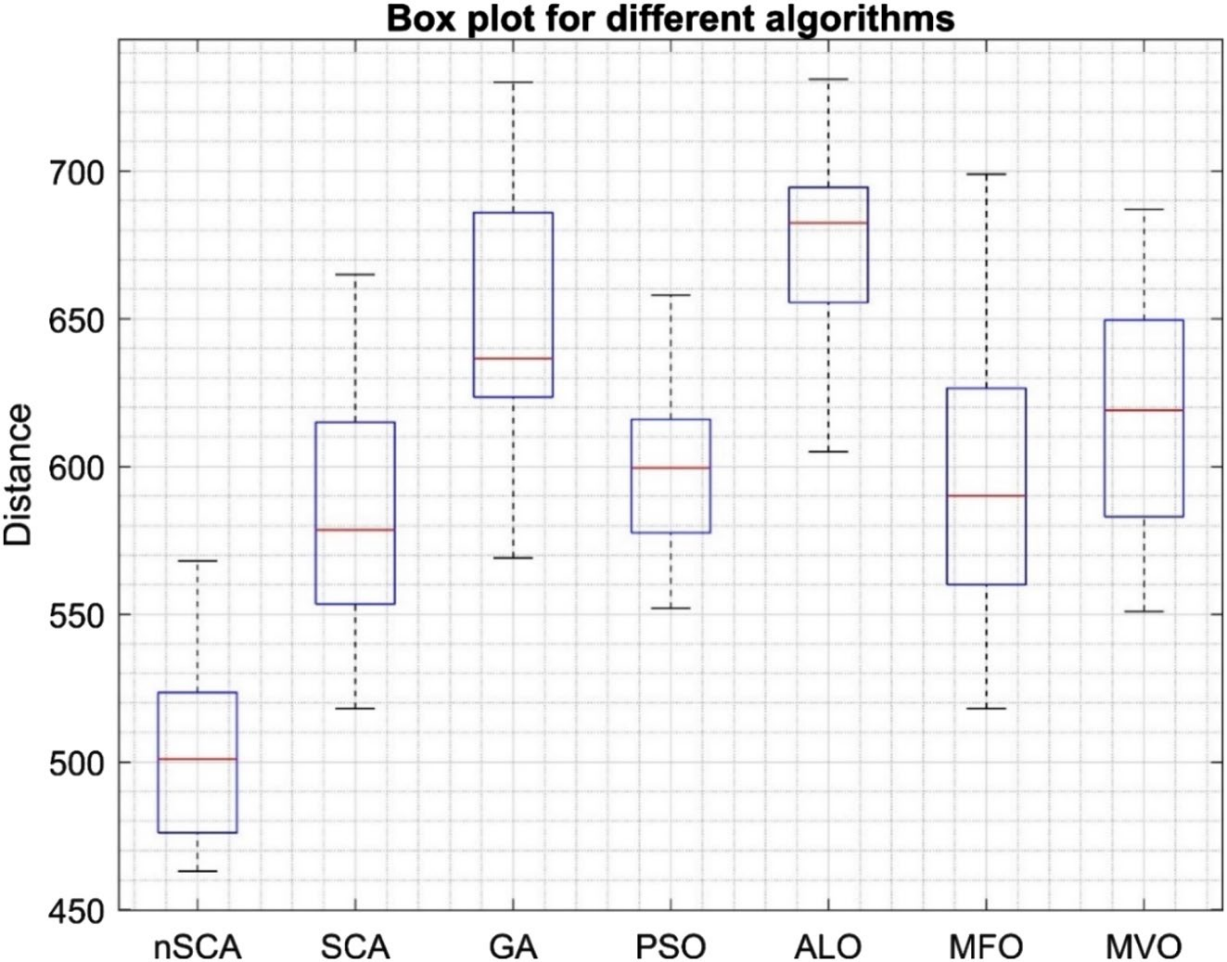
Box plot of nSCA, SCA, GA, PSO, ALO, MFO and MVO on 16-customer problem.

**Figure 12:**
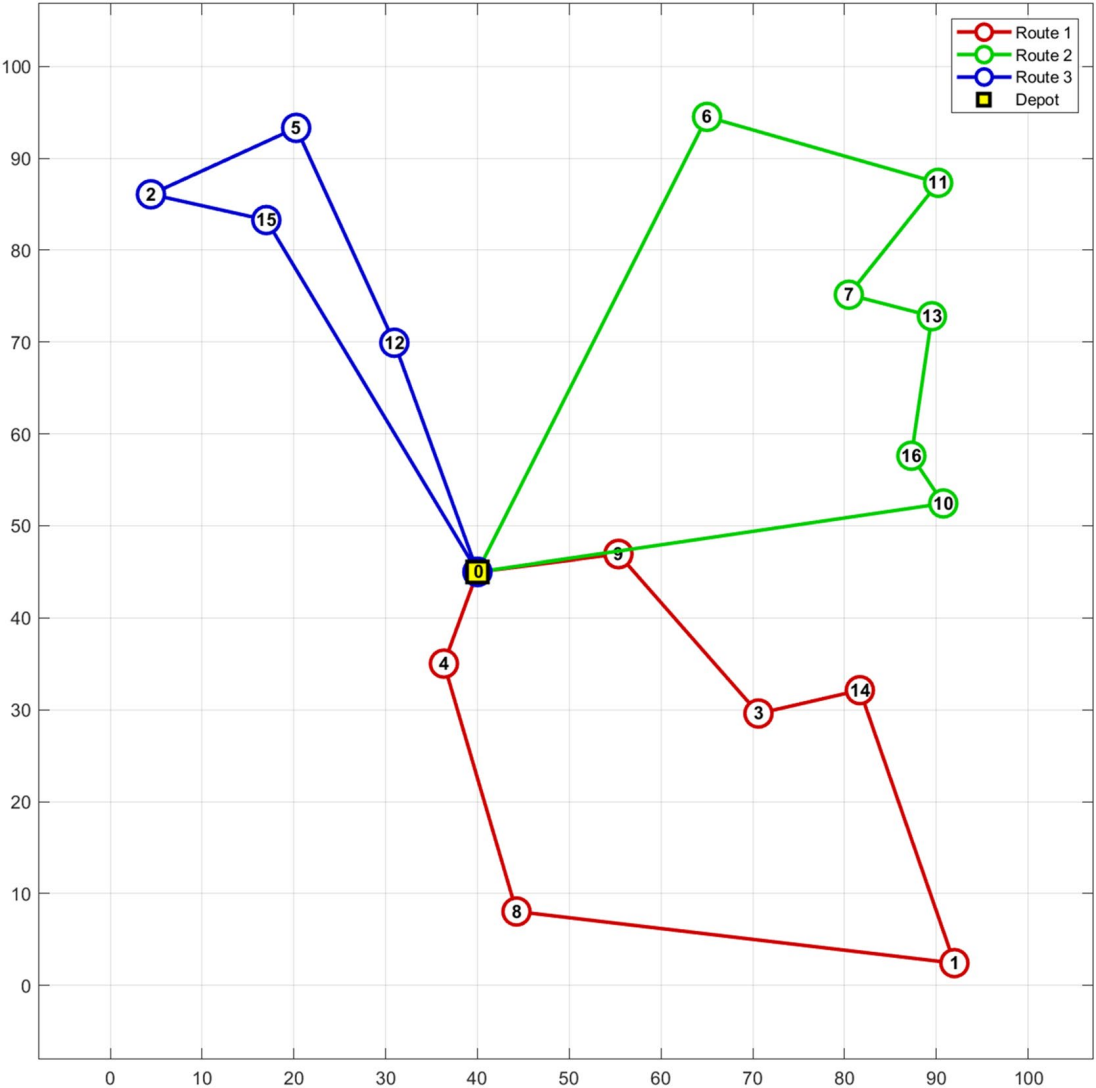
Best solution of the 16-customer problem.

Figure 11 provides additional evidence underscoring the superiority of nSCA, with its data distribution clearly outpacing other algorithms. The specific routing for the two vehicles, illustrating the optimal solution ascertained by nSCA, is delineated in Table 18. For an enriched visual perspective, this routing is further illustrated in Fig. 12.

**Table 18:** Routing of vehicles and distance obtained by nSCA on 16-customer problem.

Routes of the vehicles on 16-customer problem		Distance
Route 1	0 → Customer 4 → Customer 8 → Customer 1 → Customer 14 → Customer 3 → Customer 9 → 0	168
Route 2	0 → Customer 6 → Customer 11 → Customer 7 → Customer 13 → Customer 16 → Customer 10 → 0	171
Route 3	0 → Customer 12 → Customer 5 → Customer 2 → Customer 15 → 0	124
Total distance: 463

## Conclusion

Combining the opposition-based learning (OBL) technique with the roulette wheel selection (RWS) strategy, this research introduces nSCA, a novel approach intended to boost the exploratory capabilities of the SCA. The effectiveness of nSCA undergoes rigorous scrutiny alongside notable algorithms such as GA, PSO, MFO, ALO, MVO, and the foundational SCA, using the benchmarks provided by CEC 2019 and CEC 2021 test functions. Furthermore, the versatility and prowess of nSCA are evident in its capability to adeptly address tangible discrete and continuous optimization challenges. Insights from the analysis position nSCA above many metaheuristic methodologies, highlighting its refined ability to produce premier solutions for benchmark situations and practical optimization tasks. These findings solidify the role of nSCA as an essential instrument in the field of engineering optimization, paving the way for advanced problem-solving and decision-making techniques. With a foundation built on compelling evidence, it becomes clear that nSCA stands as a potent, trustworthy mechanism, well-suited for navigating the myriad of optimization challenges encountered in real-world scenarios.

## Limitations

The efficacy of nSCA has been substantiated by the authors through the use of contemporary benchmark test suites such as CEC 2019 and CEC 2021, and its application to various practical optimization challenges has been demonstrated. However, deeper scrutiny is still deemed necessary. Enhancement in the evaluation of the robustness of nSCA could be achieved by its integration with regression or classification techniques, notably the support vector machine (SVM). By having this integrated framework applied to real-world problems, a more intricate understanding of the adaptability and efficacy of nSCA can be obtained. Through such an exhaustive assessment, richer insight into how nSCA collaborates with established machine learning methodologies will be provided, affirming the adaptability and capability of nSCA across a wider range of challenges.

## Data Availability

The corresponding author is available to provide the data, model, or code underlying the findings of this study upon request, in accordance with reasonable conditions.

## References

[1] Holland JH (1992). Adaptation in Natural and Artificial Systems: An Introductory Analysis with Applications to Biology, Control, and Artificial Intelligence.

[2] Kennedy, J. & Eberhart, R. Particle swarm optimization. In *Proc. ICNN'95-International Conference on Neural Networks* (IEEE, 1995).

[3] Rezaei F (2023). GMO: Geometric mean optimizer for solving engineering problems. Soft Comput..

[4] Mirjalili S (2016). SCA: A sine–cosine algorithm for solving optimization problems. Knowl. Based Syst..

[5] Mirjalili S, Mirjalili SM, Hatamlou A (2016). Multi-verse optimizer: A nature-inspired algorithm for global optimization. Neural Comput. Appl..

[6] Gandomi AH (2014). Interior search algorithm (ISA): A novel approach for global optimization. ISA Trans..

[7] Mirjalili S (2015). Moth-flame optimization algorithm: A novel nature-inspired heuristic paradigm. Knowl. Based Syst..

[8] Mirjalili S (2015). The ant lion optimizer. Adv. Eng. Softw..

[9] Črepinšek M, Liu S-H, Mernik M (2013). Exploration and exploitation in evolutionary algorithms: A survey. ACM Comput. Surv..

[10] Lin L, Gen M (2009). Auto-tuning strategy for evolutionary algorithms: Balancing between exploration and exploitation. Soft Comput..

[11] Xi B (2023). LGBM-based modeling scenarios to compressive strength of recycled aggregate concrete with SHAP analysis. Mech. Adv. Mater. Struct..

[12] Zhou J (2021). Optimization of support vector machine through the use of metaheuristic algorithms in forecasting TBM advance rate. Eng. Appl. Artif. Intell..

[13] Li E (2021). Prediction of blasting mean fragment size using support vector regression combined with five optimization algorithms. J. Rock Mech. Geotech. Eng..

[14] Son PVH, NguyenDang NT (2023). Optimizing time and cost simultaneously in projects with multi-verse optimizer. Asian J. Civil Eng..

[15] Son PVH, Hieu HT (2023). Logistics model for precast concrete components using novel hybrid ant lion optimizer (ALO) algorithm. Int. J. Construct. Manag..

[16] Wolpert DH, Macready WG (1997). No free lunch theorems for optimization. IEEE Trans. Evol. Comput..

[17] Son PVH, Nguyen Dang NT (2023). Solving large-scale discrete time–cost trade-off problem using hybrid multi-verse optimizer model. Sci. Rep..

[18] Zhen L (2020). Parameter estimation of software reliability model and prediction based on hybrid wolf pack algorithm and particle swarm optimization. IEEE Access.

[19] Pham VHS, Trang NTN, Dat CQ (2023). Optimization of production schedules of multi-plants for dispatching ready-mix concrete trucks by integrating grey wolf optimizer and dragonfly algorithm. Eng. Construct. Archit. Manag..

[20] Teng, T.-C., Chiang, M.-C. & Yang, C.-S. A hybrid algorithm based on GWO and GOA for cycle traffic light timing optimization. In *2019 IEEE International Conference on Systems, Man and Cybernetics (SMC)* (IEEE, 2009).

[21] Qiao W (2020). A hybrid algorithm for carbon dioxide emissions forecasting based on improved lion swarm optimizer. J. Clean. Prod..

[22] Long W (2020). A new hybrid algorithm based on grey wolf optimizer and cuckoo search for parameter extraction of solar photovoltaic models. Energy Convers. Manag..

[23] Dhiman G, Kaur A, Bansal JC (2019). A hybrid algorithm based on particle swarm and spotted hyena optimizer for global optimization. Soft Computing for Problem Solving: SocProS 2017.

[24] Şenel FA (2019). A novel hybrid PSO–GWO algorithm for optimization problems. Eng. Comput..

[25] Zhao, Y., Zou, F. & Chen, D. A discrete sine–cosine algorithm for community detection. In *Intelligent Computing Theories and Application: 15th International Conference, ICIC 2019, Nanchang, China, August 3–6, 2019, Proceedings, Part I 15* (Springer, 2019).

[26] Banerjee, A. & Nabi, M. Re-entry trajectory optimization for space shuttle using sine-cosine algorithm. In *2017 8th International Conference on Recent Advances in Space Technologies (RAST)* (IEEE, 2017).

[27] Fatlawi, A., Vahedian, A. & Bachache, N. K. Optimal camera placement using sine-cosine algorithm. In *2018 8th International Conference on Computer and Knowledge Engineering (ICCKE)* (IEEE, 2018).

[28] Reddy KS (2018). A new binary variant of sine–cosine algorithm: Development and application to solve profit-based unit commitment problem. Arab. J. Sci. Eng..

[29] Tawhid MA, Savsani V (2019). Multi-objective sine–cosine algorithm (MO-SCA) for multi-objective engineering design problems. Neural Comput. Appl..

[30] Raut U, Mishra S, Bansal JC (2019). Power distribution network reconfiguration using an improved sine–cosine algorithm-based meta-heuristic search. Soft Computing for Problem Solving: SocProS 2017.

[31] Cheng J, Duan Z (2019). Cloud model based sine cosine algorithm for solving optimization problems. Evol. Intell..

[32] Bureerat, S. & Pholdee, N. Adaptive sine cosine algorithm integrated with differential evolution for structural damage detection. In *International Conference on Computational Science and Its Applications* (Springer, 2017).

[33] Turgut OE (2017). Thermal and economical optimization of a shell and tube evaporator using hybrid backtracking search—Sine–cosine algorithm. Arab. J. Sci. Eng..

[34] Bairathi, D. & Gopalani, D. Opposition-based sine cosine algorithm (OSCA) for training feed-forward neural networks. In *2017 13th International Conference on Signal-Image Technology & Internet-Based Systems (SITIS)* (IEEE, 2017).

[35] Qu C (2018). A modified sine–cosine algorithm based on neighborhood search and greedy levy mutation. Comput. Intell. Neurosci..

[36] Pham VHS, Nguyen VN (2023). Cement transport vehicle routing with a hybrid sine cosine optimization algorithm. Adv. Civil Eng..

[37] Abualigah L, Diabat A (2021). Advances in sine cosine algorithm: A comprehensive survey. Artif. Intell. Rev..

[38] Yu, F. *et al*. Improved roulette wheel selection-based genetic algorithm for TSP. In *2016 International Conference on Network and Information Systems for Computers (ICNISC)* (IEEE, 2016).

[39] Qian W (2018). Differential evolution algorithm with multiple mutation strategies based on roulette wheel selection. Appl. Intell..

[40] Pandey AC, Kulhari A, Shukla DS (2022). Enhancing sentiment analysis using roulette wheel selection based cuckoo search clustering method. J. Ambient Intell. Hum. Comput..

[41] Asghari K (2021). Multi-swarm and chaotic whale-particle swarm optimization algorithm with a selection method based on roulette wheel. Expert Syst..

[42] Lloyd, H. & Amos, M. Analysis of independent roulette selection in parallel ant colony optimization. In *Proc. Genetic and Evolutionary Computation Conference* (2017).

[43] Cheng Y-S (2016). A particle swarm optimization based power dispatch algorithm with roulette wheel re-distribution mechanism for equality constraint. Renew. Energy.

[44] Tizhoosh, H. R. Opposition-based learning: A new scheme for machine intelligence. In *International Conference on Computational Intelligence for Modelling, Control and Automation and International Conference on Intelligent Agents, Web Technologies and Internet Commerce (CIMCA-IAWTIC'06)* (IEEE, 2005).

[45] Wang H (2011). Enhancing particle swarm optimization using generalized opposition-based learning. Inf. Sci..

[46] Luong D-L, Tran D-H, Nguyen PT (2021). Optimizing multi-mode time–cost-quality trade-off of construction project using opposition multiple objective difference evolution. Int. J. Construct. Manag..

[47] Cheng M-Y, Tran D-H (2014). Two-phase differential evolution for the multiobjective optimization of time–cost tradeoffs in resource-constrained construction projects. IEEE Trans. Eng. Manag..

[48] Shaw B, Mukherjee V, Ghoshal S (2012). A novel opposition-based gravitational search algorithm for combined economic and emission dispatch problems of power systems. Int. J. Electr. Power Energy Syst..

[49] Ewees AA, AbdElaziz M, Houssein EH (2018). Improved grasshopper optimization algorithm using opposition-based learning. Expert Syst. Appl..

[50] Tubishat M (2020). Improved salp swarm algorithm based on opposition based learning and novel local search algorithm for feature selection. Expert Syst. Appl..

[51] Price, K. *et al*. Problem definitions and evaluation criteria for the 100-digit challenge special session and competition on single objective numerical optimization. In *Technical Report* (Nanyang Technological University, 2018).

[52] Brest, J., Maučec, M. S. & Bošković, B. Self-adaptive differential evolution algorithm with population size reduction for single objective bound-constrained optimization: Algorithm j21. In *2021 IEEE Congress on Evolutionary Computation (CEC)* (IEEE, 2021).

[53] Seyyedabbasi A, Kiani F (2023). Sand cat swarm optimization: A nature-inspired algorithm to solve global optimization problems. Eng. Comput..

[54] Ma B (2023). Running city game optimizer: A game-based metaheuristic optimization algorithm for global optimization. J. Comput. Des. Eng..

[55] Song M (2022). Modified Harris Hawks optimization algorithm with exploration factor and random walk strategy. Comput. Intell. Neurosci..

[56] Rashedi E, Nezamabadi-Pour H, Saryazdi S (2009). GSA: A gravitational search algorithm. Inf. Sci..

[57] Chickermane H, Gea HC (1996). Structural optimization using a new local approximation method. Int. J. Numer. Methods Eng..

[58] Camp CV, Farshchin M (2014). Design of space trusses using modified teaching–learning based optimization. Eng. Struct..

[59] Degertekin S (2012). Improved harmony search algorithms for sizing optimization of truss structures. Comput. Struct..

[60] Sonmez M (2011). Artificial bee colony algorithm for optimization of truss structures. Appl. Soft Comput..

[61] Lee KS, Geem ZW (2004). A new structural optimization method based on the harmony search algorithm. Comput. Struct..

[62] Schutte JF, Groenwold AA (2003). Sizing design of truss structures using particle swarms. Struct. Multidiscip. Optim..

[63] Venkayya V, Khot N, Reddy V (1969). Energy Distribution in an Optimum Structural Design.

[64] Talatahari S (2013). A multi-stage particle swarm for optimum design of truss structures. Neural Comput. Appl..

[65] Li L-J (2007). A heuristic particle swarm optimizer for optimization of pin connected structures. Comput. Struct..

[66] Kaveh A, Ghazaan MI, Bakhshpoori T (2013). An improved ray optimization algorithm for design of truss structures. Period. Polytech. Civil Eng..

[67] Camp CV, Bichon BJ (2004). Design of space trusses using ant colony optimization. J. Struct. Eng..

[68] Shahrouzi M (2020). Switching teams algorithm for sizing optimization of truss structures. Int. J. Optim. Civil Eng..

[69] Archetti C (2011). Complexity of the VRP and SDVRP. Transp. Res. C Emerg. Technol..

[70] Shan, Q. & Wang, J. Solve capacitated vehicle routing problem using hybrid chaotic particle swarm optimization. In *2013 Sixth International Symposium on Computational Intelligence and Design* (2013).

[71] Zhengchu, W. *et al*. Research in capacitated vehicle routing problem based on modified hybrid particle swarm optimization. In *2009 IEEE International Conference on Intelligent Computing and Intelligent Systems* (2009).

